# What all physicians should know about women’s health: a Delphi study

**DOI:** 10.1136/bmjph-2024-001786

**Published:** 2025-01-25

**Authors:** Merel H de Heer-Koster, Chiara Benedetto, Vesna Bjegović-Mikanović, Indre Banaitytė-Baleišienė, Mary Perdiou, Eva Gerdts, Alexandra Kautzky-Willer, Julien Mazières, Alyson McGregor, Connie Newman, Susanna Price, Jeanine Roeters van Lennep, Simona Stankevičiūtė, Iris E Sommer, Gertraud Stadler, Florence Thibaut, Karine van 't Land, Marieke Wermer, Kerry Wilbur, Fedde Scheele

**Affiliations:** 1 Amsterdam Public Health, Amsterdam UMC, Vrije Universiteit, Amsterdam, The Netherlands; 2 Department of Obstetrics and Gynaecology, University of Torino, Torino, Italy; 3 Institute of Social Medicine, Faculty of Medicine, Belgrade University, Belgrade, Serbia; 4 Hospital of Lithuanian University of Health Sciences Kauno Klinikos, Kaunas, Lithuania; 5 Patient Advisory Board, APS Europa Donna Italia, Milan, Italy; 6 Department of Clinical Science, University of Bergen, Bergen, Norway; 7 Department of Internal Medicine III, Endocrinology and Metabolism, Medical University, Vienna, Austria; 8 Toulouse University Hospital, Institut Universitaire du Cancer Toulouse Oncopole, Toulouse, France; 9 Deparment of Emergency Medicine, University of South Carolina School of Medicine, Columbia, South Carolina, USA; 10 New York University School of Medicine, New York, New York, USA; 11 Departments of Cariology and Critical Care, Royal Brompton and Harefield Hospitals, London, UK; 12 Imperial College London National Heart and Lung Institute, London, UK; 13 Department of Internal Medicine, Erasmus MC University Medical Center, Rotterdam, The Netherlands; 14 Erasmus MC Cardiovascular Institute, Rotterdam, The Netherlands; 15 Department of Psychiatry, University Medical Centre, Groningen, The Netherlands; 16 Gender in Medicine, Charité Center for Prevention, Health and Human Sciences, Charite - Universitatsmedizin Berlin, Berlin, Germany; 17 University Hospital Cochin, Institute of Psychiatry and Neurosciences, Universite Paris Cite, Paris, France; 18 Faculty of Economics and Business/Aletta Jacobs School of Public Health, Rijksuniversiteit Groningen, Groningen, The Netherlands; 19 Department of Neurology, UMCG, Groningen, The Netherlands; 20 Faculty of Pharmaceutical Sciences, The University of British Columbia, Vancouver, British Columbia, Canada; 21 Research in Education, Amsterdam UMC Location VUmc, Amsterdam, The Netherlands; 22 Athena Institute, Faculty of Earth and Life Sciences, VU University, Amsterdam, The Netherlands

**Keywords:** Education, Medical, Female, Public Health, Translational Science, Biomedical, Sex Factors

## Abstract

**Objectives:**

Over the past few decades, knowledge of women’s health regarding sex and gender differences in health has increased but transfer of these new insights into medical education and clinical practice is lagging, resulting in substandard care for women compared with men. This study aimed to reach consensus on what all physicians taking care of women should know about women’s health.

**Methods:**

A Delphi study was executed involving statements prepared by experts in women’s health across 10 medical specialties and a patient advisory board. Participants were recruited from Europe and Northern America through the experts’ networks and snowball sampling. Participants voted IN/OUT on each statement based on its perceived relevance and feasibility for general physician knowledge, regardless of specialty. The statements were ranked according to a >80% consensus in the first Delphi round and a 4-point Likert scale in the second Delphi round.

**Results:**

In the first round, 44 participants fully completed the survey. 18 statements progressed to the second round, in which four additional statements were included based on participant suggestions. In the final round, 35 responses on the 22 selected statements resulted in consensus on 18 statements of the highest importance, within the following domains: the societal position of women in health, patient perception of disease and treatment, differences in symptomatology, pharmacological considerations and the impact of the female life cycle on health and disease.

**Conclusion:**

Consensus was reached on the top priority clinical conditions and public health issues in women’s health, resulting in a list of 18 statements on women’s health that every physician caring for women should know, regardless of specialty. There was also consensus on the importance of incorporating these insights into medical education. The next step involves implementing women’s health education in medical schools, postgraduate education and continuing education for medical specialists.

**WHAT IS ALREADY KNOWN ON THIS TOPIC**

- Over the past few decades, knowledge of sex and gender differences in health issues has increased. However, transfer of these new insights into medical education and clinical practice is lagging, resulting in substandard care for women compared with men.

**WHAT THIS STUDY ADDS**

- This study resulted in a top priority ranking of 18 statements on women’s health issues and a complete list of 47 statements of importance to women’s health. The topics address five categories: the societal position of women on health, patient perceptions of disease and treatment, sex and gender differences in diagnosis and symptomatology, pharmacological considerations and the impact of the female life cycle on health and disease. Implementing these topics in medical curricula will solidify knowledge of sex and gender differences among current and future physicians

**HOW THIS STUDY MIGHT AFFECT RESEARCH, PRACTICE OR POLICY**

- The findings in this study highlight the key starting topics for incorporating women’s health issues into medical education, policy and clinical practice. This study could influence guidelines, to include topics from our statement list, or to critically assess whether women’s health is represented in guidelines. For medical education, curriculum designers can use the results of this study as a starting point to include women’s health throughout the curriculum. Policy-makers can use the results of this study and discussion section as a discussion point and reference.

## Introduction

 Physicians of various specialties can only provide optimal treatment for women if they are adequately informed about the clinical conditions and public health issues that arise from sex and gender differences between men and women. Sex and gender differences exist in disease risk factors, pathophysiology, clinical presentation, diagnostic evaluation, treatment efficacy and safety, and prognosis.^1^ These differences exist due to biological, environmental and sociocultural differences.^2^ While medical curricula have traditionally been based on the male prototype, modern curricula need to integrate knowledge of sex and gender differences between men and women. Although ‘sex’ and ‘gender’ are often used interchangeably, this is incorrect. While ‘sex’ refers to the biological and genetic differences between male and female genotypes, ‘gender’ pertains to an individual’s self-identification.^3^ This study focused on the impact of sex differences and the social context of gender on health issues, therefore, this study is applicable to all cis women, and may also, partially, apply to trans men, trans women and non-binary people. While knowledge of sex and gender differences in health issues has increased,^4^ more research in this area is needed.^5^,^7^ Furthermore, the transfer of new insights and knowledge into medical education and clinical practice is lagging.^8 9^ This lack of transfer exists in medical schools and residency programmes, and among practising physicians.^9^,^12^ Because of this lack of transfer of knowledge of women’s health, women often receive substandard care.^13^

A relative lack of attention for women’s health in medical curricula was first reported by the WHO Women’s Health Report of 1994.^14^ Recent authors acknowledged that there is still an inadequate integration of sex and gender knowledge in undergraduate and postgraduate medical education.^8 12^ The term women’s health can be defined as the sex and gender differences between men and women in health, combined with health issues that are unique to women.^15 16^ Biological factors (such as sex, age and genetics) have their influence on mechanisms of disease and health, but cultural and social factors also play a role (socioeconomic status (SES), lifestyle factors and geography).^1 17^ There are a few overarching and vital topics derived from medical textbooks and literature in women’s health, which are listed in box 1.^1 18^ Previous studies have tried to connect items of women’s health in medical education, focusing mainly on topics within their own specialty.^812 19^,^24^ Recent authors notably reviewed key terms for internal medicine, aiming to identify crucial women’s health topics for curricular inclusion.^12^ Similarly, studies have addressed sex and gender differences within oncology and cardiovascular disease for medical curricula.^19 20^ A survey among medical students showed that medical students recognise women’s health and understand the value of sex-specific and gender-specific healthcare, but the authors also concluded that current curricular offerings fall short.^8^

Box 1 **Topics of transcendent importance for women’s health**

**Multiple factors influence health and behaviour**

Health and behaviour are shaped by a myriad of factors beyond sex and gender, such as ethnicity, race, geography, age, socioeconomic status, education, culture and religion.^1 18 49^ These factors interact and potentially lead to disparities,^18 50^ underscoring the importance for healthcare providers to consider these diverse influences in patient care.

**Communication, coping and stress regulation**

Communication and coping mechanisms of women differ from those of men. Effective communication in clinical interaction is a critical aspect of the quality of given care.^1^ In clinical interactions, women tend to disclose more personal information, focus more on non-verbal communication and share more psychosocial information.^51^ In contrast, men tend to share facts and use fewer words, with little emphasis on the emotional experience of their complaints.^51 52^ These differences are also adapted in sociocultural constructs, influencing our expectations and perceptions, giving other values and meaning even if men and women use the same words.^18 53^ The observations in communication style differences are highly influenced by society and culture and these observations apply to the Western culture. The differences of communication style and biased sociocultural constructs can lead to impact on female patients, as they are less likely to be referred to specialty care and they less often receive physical examination or medical check-ups than men with the same complaints, due to the differences in clinical interaction.^54 55^ These differences are also seen in stress regulation and coping mechanisms. There are sex and gender differences in both stress responses and stress psychopathology.^56 57^ Women seem to better articulate their subjective stress than men and they use social coping strategies.^58 59^ However, men tend to have stronger physiological stress reactivity, as can be seen in higher levels of glucocorticoid hormones when exposed to a stressor.^59^,^61^ When it comes to coping, men are less likely to seek help and use more self-management strategies.^62^ It is important for physicians to consider the differences in communication, coping mechanism and stress regulation since these factors highly influence how patients experience and cope with health and disease and how they present themselves in clinical interactions.

**Pain perception**

Pain perception and stress regulation in women differ from those of men because of biopsychosocial factors. Studies have shown that women experience more intense and prolonged pain than men and that women have a higher risk of pain chronification, yet physicians are less likely to prescribe pain medication for women.^63^,^66^ The differences in pain perception between men and women can be attributed to various factors, including differences in pain pathways, hormonal influences and psychosocial factors.^18 67^ Studies note cyclical variations in pain scores throughout the month for women,^68^ and there is cellular-level variation in pain pathways influenced by testosterone, which potentially makes women more susceptible to pain.^64 69^ The psychosocial factors include communication and coping differences, as described in the prior section, as well as sociocultural beliefs about femininity and masculinity. Pain expression is generally more socially acceptable among women, which potentially leads to biased pain reporting and underestimation by healthcare workers.^67^ For example, women’s abdominal pain symptoms are often dismissed, potentially due to normalisation of dysmenorrhoea,^70 71^ Furthermore, abdominal pain is more often misdiagnosed and untreated in women compared to men.^72^,^74^ Recognising and addressing biases and variations in pain presentation is essential for healthcare providers to offer equitable pain management.

While these studies have shown that it is important to incorporate women’s health topics, so far no consensus has been reached on what is considered core knowledge in women’s health that all physicians across specialties should be aware of.^4 25^ It is vital for physicians to possess foundational knowledge about the distinctions between women compared with men for equal quality in care. The objective of this study was to reach consensus on the foundational knowledge of women’s health that is imperative for all physicians who treat women, irrespective of their specialties. This study focuses on clinical conditions and public health issues in women’s health.

## Methods

This study used a classical Delphi technique with expert-based judgements.^26^ Relevant medical specialties were identified by reviewing literature, including PubMed searches and exploration of seminal textbooks on women’s health. The included specialties are listed in table 1.

The research team comprised a steering committee, project leaders and a patient advisory board. The steering committee was composed of the secretariat, the executive researcher and the supervising researcher. The project leaders were recruited based on their reputation in the field as clinicians or scientists, as demonstrated by publications, authorship in medical textbooks concerning women’s health and through network and snowballing. The network included the steering committees’ network, as well as snowballing through the network of recruited project leaders. Project leaders were recruited with an emphasis on Western geographical diversity. The supervising researcher held personal online meetings where project leaders were briefed and where mutual expectations were aligned. For each specialty included, there were multiple project leaders working in different institutions in order to prevent personal biases. All of the project leaders were invited as coauthors and all of the project leaders participated throughout the entire process of the study and the writing process of the manuscript. The patient advisory board was formed with the assistance of the European Institute of Women’s Health.

### Patient and public involvement

The research team included a patient advisory board, consisting of members from patient organisations. The patient advisory board was recruited by reaching out to the European Institute of Women’s Health, an umbrella organisation of patient organisations across Europe. They shared our call for participation in the study with their members. There was a separate information letter about the study, in comprehendible and layman English. An online meeting was held with respondents to our call to establish the patient advisory board. During this online meeting, the steering committee provided a briefing where mutual expectations were aligned. The patient advisory board was involved from the start of the study, in the design of the voters list of the Delphi process, in the interpretation of the study findings and in the writing process of the manuscript. A linguist, in collaboration with a medical doctor from the steering committee, developed layperson-friendly explanations of the statements formulated by the experts. The patient advisory board provided feedback on these explanations and was also given the opportunity to suggest additional topics for the statement list if deemed necessary. The steering committee provided updates to the patient advisory board throughout the study and members of the patient advisory board were invited to participate in the writing process of the manuscript as coauthor. The final article will be shared with the patient advisory board. A layman summary of the article will be provided for dissemination among the patient organisations members and the results of this study may be used for patient education materials.

### Voting list

The voting list consisted of clinical conditions and public health topics in women’s health. It was composed based on literature and refined by suggestions of the project leaders and the patient advisory board. First, the steering committee established guidelines for statement formulation to ensure consistency and academic rigour. Next, the project leaders drafted statements on topics that they deemed essential for inclusion in the voting list. These statements were reviewed by the patient advisory board, which added any statements they felt were missing. The statements were reviewed by the steering committee for duplicates and literature context, followed by a linguistic review for consistency, precision, and avoidance of ambiguity. The supervising researcher piloted the voting list for final approval. The list was subsequently distributed to participants via Qualtrics, with literature references per statement available for the voters. Participants could suggest adding topics after the first round if they felt topics were missing.

### Participants

Project leaders and the steering committee invited medical specialists within their networks who were either knowledgeable about or deeply interested in women’s health to participate in the Delphi rounds. There were no restrictions on the type of specialty of the participants. The determination of the sample size was informed by literature, which suggests that a sample size of 30–50 participants is optimal for Delphi studies, depending on the complexity of the problem, the heterogeneity of the panel and resource availability.^27^,^29^ To account for the anticipated attrition commonly observed across Delphi rounds, 100 participants were initially targeted for invitation.^30^ Each participant who was willing to take part in the study was provided in advance with a participant information letter, a consent form, and a briefing outlining the two Delphi rounds. Only those who provided written informed consent were included in the study. The target for inclusion was set at 100 participants for the voting process. Responses were anonymised and processed in Qualtrics.

### Delphi process and data collection

**Figure 1:**
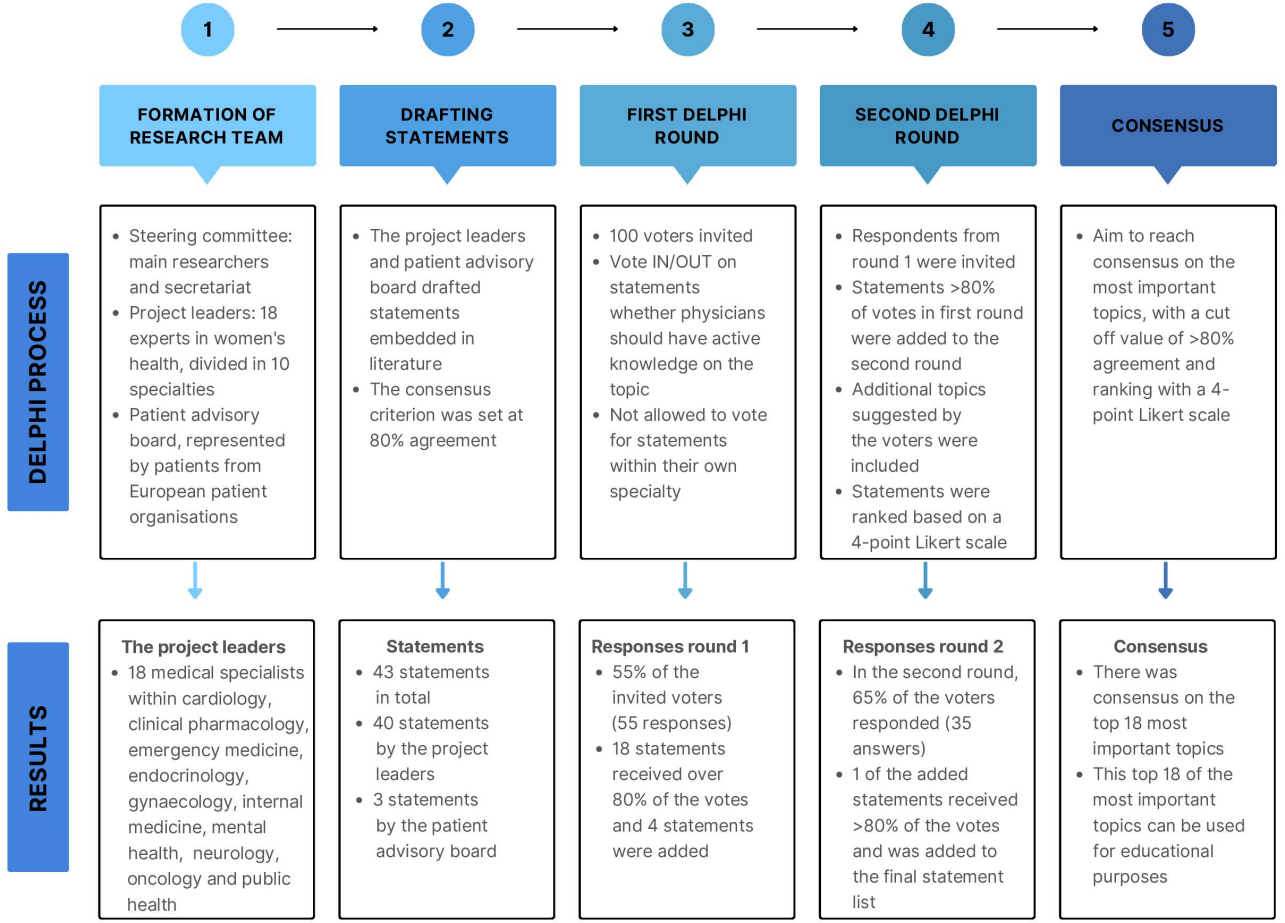
Delphi process.

The Delphi method applied in this study is categorised as classical based on five characteristics.^26^ Participants engaged in anonymous surveys using a standardised questionnaire in each round. Responses underwent univariable analysis, and consent criteria were predefined. The study comprised two voting rounds. Voter feedback influenced adjustments for the subsequent round; likewise, the steering committee provided feedback to voters before the second round. The Delphi process is illustrated in figure 1.

The participants voted on whether they thought a topic to be core knowledge in women’s health clinical conditions and public health issues. To avoid unconscious bias, they were not allowed to vote on statements in their own specialty. This approach was adopted to avoid the potential influence of their professional perspective, which might lead to an implicit prioritisation of topics related to their specific area of expertise.

For the first round, participants were asked to vote IN/OUT based on the following question:

Please ask yourself if you believe it is not only desirable, but also reasonable and feasible that physicians of all specialties should have active knowledge about the topic covered in the statement.

The consensus criteria in this Delphi study were predetermined and described in the Delphi process. A strict cut-off value of 80% was applied. This threshold was determined based on recommendations from the literature and discussions with the project leaders, ensuring methodological rigour.^28 31 32^ Recommendations in the literature advise to set a cut-off value between 70% and 80%.^28 31 32^ Prior to the start of the Delphi rounds, the project leaders emphasised that all topics are inherently important. However, given the study’s objective to identify a core set of topics for all physicians regardless of specialty and to establish a starting point for curricular content, a stringent threshold was adopted for advancing statements to the next round.

Statements failing to achieve an 80% vote threshold were excluded, with the remainder advancing to the next round. Participants could provide overall feedback, propose new topics or modifications to existing statements, which were evaluated by the steering committee and incorporated into the second round.

The second Delphi round served two purposes: first, to allow participants to provide feedback and suggest new topics or modifications to existing ones; and second, to establish a priority ranking for the top results. While all statements were considered to be highly important, practical constraints often require a phased approach. The priority ranking was intended to provide clarity and actionable insights as a starting point for improvement on women’s health education. The participants first voted IN/OUT on the newly added statements. Next, the importance of all the advanced statements was ranked using a 4-point Likert scale, from low (1) to high (4) importance. Statements were ranked based on the total sum of the Likert scale points. The statements were analysed and ranked using Qualtrics Analysis and Excel, resulting in a definitive list of core knowledge in women’s health.

Participation was voluntary and there were no forms of compensation provided. Access to data was limited to the steering committee, with anonymity safeguarded by means of Qualtrics, which anonymised incoming data by omitting names, email addresses and IP addresses. Withdrawal from the study did not permit the removal of previously submitted data due to their anonymous processing and their potential prior inclusion in consensus outcomes. This study was reported in line with the Standards for Quality Improvement Reporting Excellence (SQUIRE) guideline and the ACcurate Consensus Reporting Document (ACCORD) guideline.

## Results

### Design and research team

The results of this Delphi process are described in figure 1. The research team comprised 18 project leaders in 10 medical specialties. The geographical spread of the project leaders and the patient advisory board members covered 13 countries, including the UK, Scandinavia, Europe, the USA and Canada.

### Voting list and voters

The project leaders drafted 47 statements that they considered essential for the core knowledge of women’s health. They based the selection of these statements on literature and on their expertise in women’s health. The patient advisory board added three more statements to this list. 7 statements were merged or removed due to duplication, resulting in a list of 43 statements in the voting list for the first round. The topics of the 43 statements are listed in table 1, and the complete list of statements is included in online supplemental appendices. The research team invited 100 voters, once again with a wide geographical spread across Europe, Canada and the USA.

**Table 1:** Topics suggested by project leaders and patient advisory board

Specialties in alphabetical order	Topics
**Medical experts**	
Cardiology	Cardiac damage
	Hypertension*
	Hypertensive disorder of pregnancy
	Inflammatory comorbidities
	Non-obstructive coronary artery disease
Clinical pharmacology	Treatment considerations for nursing women*
	Treatment considerations for pregnant women*
	Treatment considerations for postmenopausal women*
	Treatment considerations for women intending to become pregnant*
Emergency medicine	Acute cardiovascular disease
	Role of oestrogen
	Sex differences in medical training
	Symptomatology bias: symptoms in women are not atypical*
	Unconscious bias and misdiagnosis in cardiovascular disease*
Endocrinology	Diabetes and obesity
	Hypercholesterolaemia
	Osteoporosis*
	Thyroid disease
Gynaecology	Contraception and prevention of STD
	Endometriosis
	Preconception counselling
	Recognition of menopausal issues*
	Safeguarding sexual and reproductive rights*
Mental health	Depression and reproductive events
	Diagnosis and mental healthcare after sexual violence, rape, drugs
	Effects of early exposure to child pornography
	Psychopharmacotherapy, importance of sex and gender differences
Neurology	Cerebral vein thrombosis
	Migraine
	Stroke
	Treatment of epilepsy with valproic acid in women of childbearing age
Oncology	Addressing the bias in symptoms in women in the field of oncology*
	Efficacy and toxicity of cancer treatments in women
	Importance of screening in prevention of malignant diseases in women*
	Non sex related cancer in women, sex differences
	Preclinical and translational research to address specific differences in cancer in women (excluding breast cancer, gynaecological cancers)
Public health	Community participation in development of reproductive health programmes and services
	Reproductive and well-childcare services as essential to health of women and the population*
	Socioeconomic inequalities and women’s health*
	Violence against women*
**Patient advisory board**	Awareness and guidance for fertility preservation during treatment*
	Awareness and guidance for women to adapt to body changes due to disease and treatment*
	Psychological support for the influence of disease: physically, emotionally and regarding family bonds*
**Added statements (suggested by participants)**	
Anaesthesia/surgery	Peri-operative care
Internal medicine	Autoimmune disease in women*
Neurology	Insomnia
Urology	Urology

The complete list of statements and references can be found in the online supplemental appendices.

* Indicates statements in the top 18.

### Delphi process

Data collection took place between July and December 2023.

In the first round, 55 (55%) of the invited voters responded. Other invited voters were unresponsive without giving a reason, despite previous agreement of the voters to participate and efforts of the steering team in sending reminders. Of the 55 respondents, 44 completed the questionnaire. Of the 11 respondents with incomplete questionnaires, 4 were excluded because they had completed less than half of the survey.

Of the 43 statements in the voting list, 18 received >80% of the votes and were, therefore, advanced to the subsequent round. Voters suggested several alterations to the existing statements, and they suggested four additional topics for statements. These alterations and additions were reviewed and processed by the steering committee.

In the second and final round, the 55 respondents from round 1 were reinvited, with the limitation of anonymity preventing confirmation of complete survey submissions of the previous round. Of the 55 invited voters, 35 (64%) answered, all of whom had completed the questionnaire. The other 20 invited voters were unresponsive to reminders, which were sent to all voters since answers were processed anonymously.

The four statements introduced after the first round underwent the IN/OUT voting process, and one of these statements achieved the requisite 80% approval for inclusion. The prioritisation of the 18 statements from the first round and the newly approved statement from the second round was conducted using a 4-point Likert scale, resulting in a hierarchical ranking based on the highest sum of points of the Likert scale. One statement was removed after the second round, based on feedback of the voters, since the statement did not concern a specific clinical condition or public health item. This statement concerned the imperative to integrate sex and gender differences comprehensively into medical training curricula. However, this statement received 88% of the votes. Therefore, this statement is mentioned as an individual finding.

### Consensus

After the second round, consensus was reached. The criteria for consensus were fulfilled based on a combination of voting percentages exceeding 80% and priority rankings. The final list of topics comprised 18 items, which are summarised in table 2.

**Table 2:** Priority ranking of top 18 clinical conditions and public health issues in women’s health

Topics		Points of max. possible score (140)*	
1	Gynaecology: Safeguarding sexual and reproductive rights	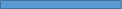	132
2	Emergency medicine: Unconscious bias and misdiagnosis in cardiovascular disease	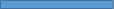	130
3	Emergency medicine: Symptomatology bias, symptoms in women are not ‘atypical’	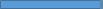	127
4	Clinical pharmacology: Treatment considerations for pregnant or nursing women	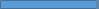	126
5	Public health (including Mental Health): Violence against women	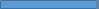	126
6	Cardiology: Hypertension	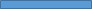	124
7	Gynaecology: Recognition of menopausal issues	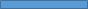	123
8	Clinical pharmacology: Treatment considerations for women intending to become pregnant	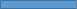	120
9	Public health: Socioeconomic inequalities and women’s health	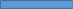	119
10	Endocrinology: Osteoporosis	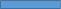	116
11	Public health: Reproductive and childcare services for improving the health of women and of the population	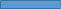	116
12	Oncology: The bias regarding symptoms in women in the field of oncology	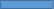	114
13	Patient advisory board: Awareness and guidance of fertility preservation during treatment	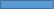	114
14	Public health: Importance of screening in the prevention of malignant diseases in women	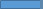	110
15	Internal medicine: Autoimmune disease in women	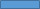	102
16	Patient advisory board: Psychological support for the influence of disease: physical, emotional and regarding family bonds	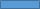	102
17	Clinical pharmacology: Treatment considerations for postmenopausal women	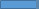	101
18	Patient advisory board: Awareness and guidance to adapt to changes in the body due to disease and treatment	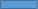	97

The overall topics address five categories: the societal position of women on health, patient perceptions of disease and treatment, differences in diagnosis and symptomatology, pharmacological considerations and the impact of the female life cycle on health and disease.

* The points refer to the maximum possible score of the Likert scale scores in round 2 (max 140 points), all these statements received >80% of consensus votes in round 1.

## Discussion

This study aimed to establish consensus on the fundamental clinical conditions and public health issues pertinent to women’s health that should be common knowledge for all physicians who treat women, regardless of their specialty. After two rounds of voting, consensus was reached and statements were successfully prioritised. In the analysis of the top 18 statements, several overarching domains emerged: the societal position of women on health, patient perceptions of disease and treatment, differences in diagnosis and symptomatology, pharmacological considerations, and the impact of the female life cycle on health and disease.

### Perspectives

The discourse on women’s health encompasses various perspectives. Our study endeavours to contribute to these discussions, with the potential of the findings to inform medical education, patient education and advocacy efforts. We explore these applications in the sections that follow.

### Medical education

The outcomes of this study are intended to generate educational materials for medical curricula, to incorporate the identified clinical conditions and public health issues as essential components of women’s health knowledge. A common barrier to the integration of new content into medical curricula is the perceived lack of space within already comprehensive programmes.^33 34^ However, the topics discussed in this article are not entirely new since they are often already part of the medical curriculum from a male-centric point of view in medicine. Most women’s health topics discussed in this article can be dealt with through additions to and modifications of existing curricular content. We advocate the inclusion of women’s health as a recurring theme throughout medical education rather than as a standalone course, which could convey the message that women’s healthcare is a distinct entity rather than an integral component of the standard care provided to half of the population. The full list of statements provided in online supplemental appendices could serve as the basis for more extensive education in women’s health.

### Patient education

The contributions of the patient advisory board were invaluable to this study; from their perspective, they added topics to the statement list. With these contributions, they underscored the importance of patient-centred care, which emphasises shared decision-making and patient feedback as cornerstones of modern healthcare practices.^35 36^

The need for knowledge of women’s health is not limited to medical education but is also relevant for the general society and for patient education.^37^ Recognition of health issues by patients themselves is the first step towards healthcare access and, therefore, it is important to further improve patient education. There are several domains, such as menopause and menstruation, in which societal norms do not recognise pathophysiology or in which complaints are normalised.^38^,^41^ We should not only increase knowledge of sex and gender differences in medical education but also develop material for patient education since access to information is an important precondition in patient education.^42^

### Advocacy and politics

Ever since 1994, various organisations, including the United Nations and WHO, have been advocating improved access to and quality of care for women.^1443^,^46^ Advocacy and political action are crucial for advancing women’s health, primarily through raising awareness, policy development, research funding, embracing intersectionality and consideration of economic impact. To further advance women’s health, it is not only essential to raise awareness among healthcare professionals and the public but also among policy-makers. There is a pressing need for policy adjustments and for the formulation of specific women’s health strategies at global, continental and national levels. Research funding is vital for sustained investigation in this field. Recognising and addressing intersectionality is necessary to ensure inclusivity and equality in health policies. The economic implications of healthcare inequalities between men and women also warrant consideration.^47^

### Limitations and future research

There are some important limitations associated with our study. First, the findings in this study highlight the key starting topics for incorporating women’s health issues into medical education. The entirety of the domain of women’s health itself naturally surpasses the scope of this study.

Second, this study is limited to sex and gender differences in women’s health, whereas health is also influenced by other biological, cultural and social factors. In addition, sex and gender differences in medicine apply to both men and women. For example, in the UK, there is the ’Glasgow effect’, where males with a lower SES have a lower life expectancy, with a mean around 55 years.^48^ This lower life expectancy might be due to hard physical labour, combined with an unhealthy lifestyle due to a lower SES.^18^ A lower SES influences health due to limited finances, less education on healthy habits and social influences that stimulate unhealthy habits such as smoking and alcohol abuse.^18^ Future research could explore women’s health topics in relation to other factors or address specific sex and gender issues for men in medical education.

Third, the scope of this study encompassed the context of Western countries in Europe and North America. In view of the impact of social and cultural factors on health, the topics for education on women’s health might differ in other geographical areas or contexts. Future studies might employ our approach to identify these topics in other geographical areas.

Lastly, there are limitations associated with the Delphi method. This method was well suited to our study aim of finding consensus among women’s health experts. While the Delphi method effectively facilitated consensus among experts in women’s health, it is inherently based on expert opinions. To achieve academic rigour to the fullest extent, we solicited input from two experts in each specialty, enlisted the assistance of a language expert to review the text, incorporated the patient perspective by inviting an umbrella organisation for a wide variety of patient organisation within women’s health and reduced the risk of bias by preventing voters to vote within their own area of expertise. Despite these efforts, a potential disadvantage of the Delphi method in medical research is the risk of group bias or limited representativeness by focusing on clinicians and public health specialists in the combined expert opinion. This may have resulted in missing topics that other specialists in the field would have deemed important knowledge for women’s health. This may also apply to the patient advisory board, as they bring in their personal perspectives and experiences. Their suggestions may have been influenced by their personal patient journeys, which brings an essential perspective on one hand, while also potentially leading to limited representativeness.

Future research could explore other avenues to solidify women’s health knowledge as a fundamental aspect of medical education, addressing the persistence of knowledge gaps in clinical practice and education. The current lack of transfer of knowledge in women’s health raises questions about why this knowledge is not yet considered fundamental and why limited action has been taken in the clinic. Research on sex and gender differences should continue and medical curricula should be continuously updated on new insights.

### Recommendations on the use of study results

1. Starting point for medical education
  The identified core topics and their ranking provide a concise starting point for integrating women’s health into medical education. These topics can be linked to existing curricular content and the ranking offers a concise starting point and guide for prioritising for those seeking a more focused starting point. Addressing curriculum gaps requires a shift away from viewing women’s health as solely focused on the female reproductive system. While most medical curricula already include sections on women’s reproductive health, significant gaps also lie in areas outside this scope. The identified core topics aim to bridge these gaps and promote a more comprehensive integration of women’s health into medical education.
2. Adaptation and expansion
  Beyond the starting point, the statement list can be expanded and tailored to fit specific institutional, national or regional contexts. It also serves as a reflective tool, offering a full statement list with important topics and examples of how women’s health can be adapted to meet local needs.
3. Clinical guidelines
  The statement list can function as a checklist or example for evaluating whether existing and newly written clinical guidelines adequately address women’s health issues. The complete list, with its accompanying references to relevant literature, can serve as both a reference and an example for integrating evidence-based women’s health content into guidelines.
4. Patient education, policy and stakeholder collaboration
  To support patient education, policy development and stakeholder collaboration, the study outcomes will be summarised in lay terms. The summary will be shared with patient organisations via the European Institute of Women’s Health and Europa Donna, whom we collaborated with in this study. European Non Governmental Organisations (NGO), such as C4EB, and other national organisations will also be approached to help disseminate the findings. The findings are relevant for national and European-level policy discussions, offering a start for shaping education, patient information and clinical guidelines.

## Conclusion

The 18 core clinical conditions and public health issues in women’s health in Europe and North America have been identified based on consensus with a cut-off value of 80% and priority ranking. This knowledge, along with the general acknowledgements, should be incorporated into medical education. Implementing these topics in medical curricula will solidify current and future physicians’ knowledge of sex and gender differences that are essential for women’s health, which is imperative to ensure that women no longer receive substandard care.

## Supplementary material

10.1136/bmjph-2024-001786 online supplemental file 1

## Data Availability

Data are available on reasonable request. All data relevant to the study are included in the article or uploaded as supplementary information.
