# Supplementary material for: What all physicians should know about women’s health: a Delphi study
Source: BMJ Public Health. 2025 Jan 25;3(1):e001786. doi: 10.1136/bmjph-2024-001786 (PMC12320051; doi:10.1136/bmjph-2024-001786)
Supplement: online supplemental file 1 [file bmjph-3-1-s001.docx]

# Appendices

Complete statement and voting list

1. **Statements: Gynaecology**

| **No** | **Area of competence** | **Statement** |
| --- | --- | --- |
| 1 | Safeguarding sexual and reproductive rights | Sexual and reproductive health and rights, which are related to bodily autonomy and self-determination in sexuality and reproduction, are particularly relevant to gender equality, but they also concern every individual. Nowadays, those rights are still infringed even in developed countries. Indeed, globally, 1 in 3 women aged 15 to 49 have experienced physical and/or sexual violence at least once in their lifetime*.* Sexual violence has serious consequences on women’s physical, mental, sexual and reproductive health. Negative effects can include acute injuries, unintended pregnancies, sexually transmitted infections, chronic pain, gastrointestinal illness, gynaecological problems, substance abuse, depression and even suicide*.*  As to sexual health, it is known that sexual dysfunction in adults, which can be treated successfully, occurs in about 40% of women and in some cases can be associated with child sexual abuse. For all these reasons, medical professionals working with female patients should be knowledgeable and sensitive to signs of sexual dysfunction and/or sexual violence, using specific questionnaires if necessary, to identify the type of problem and to be able to refer affected women to the specific healthcare providers they may need to improve their quality of life. |
| 2 | Contraception and prevention of STD | According to a 2022 report by UNFPA, nearly half of all pregnancies woldwide, at a total of 121 million every year, are unintended, and over 60 percent of these pregnancies end in abortion. The key drivers of unintended pregnancy are gender inequality and limited knowledge and awareness of family planning, often in cases of conflicts, crises, or in adolescent girls. Numbers of unintended pregnancies vary according to region, country, levels of income and education. For example, in Europe, the prevalence of teenage pregnancy ranges from 17/1000 in the South to 41/1000 in the East (ref: Fedde). Indeed, investing in family planning is a very cost-effective public health intervention, due to its high returns. Besides, effective contraception is the best way to prevent abortion. Moreover, hormonal contraception has numerous other positive effects, including a reduction in debilitating menstrual pain and in problems related to endometriosis. |
| 3 | Focus on pre-conceptional besides prenatal counselling | Most women in the Western world will use prenatal care. However, it is less common to provide counselling to women who are not yet pregnant but may wish to conceive. In most medical specialties, there are examples of diseases and treatments that warrant both preconceptional counselling and preventive action. Examples are the use of teratogenic drugs, diabetes, thyroid disease and psychiatric disorders. Each doctor working with female patients in the fertile years must keep a future pregnancy in mind. |
| 4 | Recognition of endometriosis and debilitating menstrual pain | Doctors often misdiagnose endometriosis. Women may expect a delay in diagnosis of 4 to 11 years , whilst it can cause serious menstrual pain and infertility. Overall, 2 to 29% of women suffer from severe menstrual pains. These pains impact both quality of life and the ability to function during menstruation. About a quarter of women report heavy menstrual bleeding. Whether debilitating menstrual pain and heavy bleedings are due to endometriosis or to another cause, medical interventions are often favourable. Each doctor working with women must be sensitive to debilitating menstruation, refuse to regard such events as normal and advise treatment. |
| 5 | Recognition of menopausal issues | The menopause comes with physical and mental complaints in more than 50% of women. In 10% of women, menopause may seriously disturb function. Treatments are usually available and effective. Since menopausal complaints vary widely and differ between women, it can be difficult for doctors to recognise menopause as the cause. Each doctor working with women must be sensitive to the role of menopause when making a diagnosis, resist the normalisation of menopausal issues and advise treatment. |

**Gynaecology References**

| **Statement** | **References** |
| --- | --- |
|  | - Innovative Approaches to Sexual and Reproductive Health and Rights Report: Committee on Equality and Non-discriminations: Council of Europe Parliamentary  Documents 15248: 23 January 2023. - WHO 2018: Violence Against Women Global Estimates: WHO/SRH/21.6 World Health Organization 2021. - Violence Against Women and Girls: What The Data Tell Us: World Bank 2022. - Female Sexual Dysfunction: Pharmacologic and Therapeutic Interventions Lindsay J Wheele et al Obstet Gynecol 2020 Jul;136(1):174-186. - Trauma Violence Abuse, 2022 Aug 20: The Relationship Between Child Sexual Abuse and Sexual Dysfunction in Adults: A Meta-Analysis:  [Shao-Jie Wang](https://pubmed.ncbi.nlm.nih.gov/?term=Wang+SJ&cauthor_id=35993405)[https://pubmed.ncbi.nlm.nih.gov/35993405/](https://pubmed.ncbi.nlm.nih.gov/35993405/#full-view-affiliation-1) et al)( Laumann et al., JAMA). - UNFPA Report, <https://news.un.org/en/story/2022/03/1115062>. - Coulson J. et al, China Population and Development Studies (2023) 7:1–14, <https://doi.org/10.1007/s42379-023-00130-7>. - 2023- <https://www.uptodate.com/contents/image/print?imageKey=OBGYN%2F119763>. |
|  |  |

1. **Statements: Mental Health**

| **No** | **Area of competence** | **Statement** |
| --- | --- | --- |
| 1 | Sex and gender matter in psychopharmacotherapy | Women and men should not receive the same medication dosage. Due to differences in pharmacokinetics and pharmacodynamics, psychiatric medication is often relatively overdosed in women compared to men. This deviation results in more side effects, more medical consultations and, often, a woman’s decision to discontinue treatment due to the side effects of relative overdosage. Preclinical and clinical research is usually  conducted in male participants, and gender-based clinical research is still missing, as are gender-based guidelines for prescription. Moreover, in the vast majority of clinical studies, pregnant or breastfeeding women are excluded, which results in limited evidence of medication safety and efficacy in this population, even though a significant percentage of women receive psychotropic drug treatment during pregnancy or breastfeeding. |
| 2 | Depression and reproductive events | Female hormones play a crucial role in women's mental health. Although the oral contraceptive pill has been used worldwide by millions of women for over 50 years, we cannot predict which women are likely to experience adverse effects on mood, nor which formulations are more likely to be responsible for these effects.  Up to 80% of women experience some challenges relating to menses, 40% have premenstrual symptoms, and 10 to 15% have premenstrual dysphoric disorder, which may result in suicide ideation. There are no treatment guidelines. In women aged 42 to 52, depression rates increase up to 16 times, and most of these women do not receive adequate care.  Finally, 1 in 5 mothers of children aged 24 months or younger are diagnosed with depression, which may result in neurodevelopmental consequences in their babies.  To offer adequate care, screening for early diagnosis is needed, as is pharmacological research. |
| 3 | Optimal diagnosis and mental healthcare after sexual violence, rape drugs | Women are seven times more likely to become victims of sexual violence than men (Statistics Canada, 2017). Sexual violence manifests as rape, be it within marriage or dating relationships, by strangers, or during armed conflict, as unwanted sexual advances, or as sexual harassment. Sexual violence also includes forced marriage or cohabitation, denial of the right to use contraception or measures to protect against sexually transmitted diseases, and forced abortion. Coercion includes the use of physical force and may involve psychological intimidation. It can also occur when a person is unable to give consent, e.g., while drunk, drugged, asleep, too young or mentally incapable of understanding the situation. Assaults are significantly under-reported, with some studies stating that between 80 and 90% of victims never disclose abuse.  Sexual assault is a traumatic experience that can have long-term physical and psychological effects, including post-traumatic stress disorder (50% of cases) and the triggering or exacerbation of serious mental health disorders, substance use issues (riskX26) or attempted suicide (riskX13).  Screening should be increased in the general population to enable early diagnosis of sexual violence and to offer gender-specific care in order to reduce the risk of psychological sequels. |
| 4 | Effects of (early) exposure to (violent) pornography | The average age of first pornography exposure is between 11 and 12 years. Since the brains of teenagers are especially plastic, early use of sexually explicit material may harm their ability to relate to real life and may increase the risk of sexual addiction and sexual violence. Longitudinal studies are needed, and governments should improve sexual education of children and teens and help protecting children against early porn exposure. |

**Mental Health References**

| **Statement** | **References** |
| --- | --- |
| 1 | Why sex and gender matter in health research synthesis. http://methods.cochrane.org/equity/sex-andgender-analysis  Draft report on promoting gender equality in mental health and clinical research; Committee on Women’s Rights and gender Equality; 2016/2096 (INI)) European Parliament.  Robiyanto, R., Schuiling-Veninga, C.C.M., Bos, J.H.J. et al. Exposure to psychotropic drugs before and during pregnancy: what has changed over the last two decades?. Arch Women's Ment Health 26, 39–48 (2023). https://doi.org/10.1007/s00737-023-01290-8 |
| 2 | <https://www.maprc.org.au/sites/www.maprc.org.au/files/Beattie%20Smith%20Lecture%20May%202019_Online.pdf> |
| 3 | Hughes, E., Lucock, M., & Brooker, C. (2019). Sexual violence and mental health services: A call to action. Epidemiology and Psychiatric Sciences, 28(6), 594-597. doi:10.1017/S2045796019000040. |
| 4 | - Smahel, D., Machackova, H., Mascheroni, G., Dedkova, L., Staksrud, E., Ólafsson, K., Livingstone, S., and Hasebrink, U. (2020). EU Kids Online 2020: Survey results from 19 countries. EU Kids Online. Doi:10.21953/lse.47fdeqj01ofo EU Kids Online 2020: Survey results from 19 countries. |

1. **Statements: Cardiology**

| **No** | **Area of competence** | **Statement** |
| --- | --- | --- |
| 1 | Hypertension | Hypertension is world-wide the greatest risk factor for mortality in women. Growing evidence shows that end organ damage is more severe in women than in men at similar levels of blood pressure.  The risk for CVD (cardiovascular diseases) increases at a lower blood pressure level in women than in men. In 40-year old women, a high-normal blood pressure is associated with a 2-fold higher risk for myocardial infarction before the age of 60 compared to normal blood pressure (<130/80 mmHg). This association is not found in men. The findings suggest that sex-specific definitions of hypertension should be developed. |
| 2 | Hypertensive pregnancy disorder | Women are at the highest risk to develop premature hypertension after severe hypertensive pregnancy disorders (preeclampsia/HELLP). After these pregnancies, women should be advised regular blood pressure measurements and/or home blood pressure measurements. |
| 3 | Inflammatory co-morbidities | Women with inflammatory co-morbidities (thyroid disorders, rheumatic disorders, fibromyalgia) are at higher risk for CVD. These co-morbidities should be accounted for in risk estimation at middle age. Doctors should know that women with inflammatory diagnosis should receive risk estimation at middle age, all physicians should know about the increased risk for CVD in these women. |
| 4 | Non-obstructive coronary artery disease | Non-obstructive coronary artery disease (CAD) is twice as prevalent in women compared to men. In symptomatic women (< 75 years) without obstructive CAD an invasive coronary function test should be done to assess the appropriate diagnosis Doctors should be aware of the higher prevalence in women in non-obstructive CAD. |
| 5 | Cardiac damage | At least 15% of women who are treated with radiotherapy and chemotherapy for breast cancer will develop cardiac damage (CAD and/or heart failure) during treatment or many years afterwards. It is therefore recommended to develop a cardio-oncology service in each clinic to monitor those women who are at highest risk. Every physician should be aware of these risks of complications or late side effects  and consider the possibility of cardiac damage in symptomatic patient with a history of radio- or chemotherapy. |

**Cardiology References**

| **Statement** | **References** |
| --- | --- |
| 1 | - Gerdts E, et al. Sex differences in arterial hypertension. Eur Heart J. 2022 Dec 7;43(46):4777-4788. |
| 2 | - Muijsers HEC, Wu P, et al. Home blood pressure monitoring detects unrevealed hypertension in women with a history of preeclampsia: Results of the BP-PRESELF study. Am J Prev Cardiol. 2022 Nov 11;12:100429. |
| 3 | - Konst RE, et al. The pathogenic role of coronary microvascular dysfunction in the setting of other cardiac or systemic conditions. Cardiovasc Res. 2020 Mar 1;116(4):817-828. |
| 4 | - Kunadian V, Chieffo A, Camici PG, et al. An EAPCI Expert Consensus Document on Ischaemia with Non-Obstructive Coronary Arteries in Collaboration with European Society of Cardiology Working Group on Coronary Pathophysiology & Microcirculation Endorsed by Coronary Vasomotor Disorders International Study Group. Eur Heart J. 2020 Oct 1;41(37):3504-3520. |
| 5 | - Lyon AR et al. 2022 ESC Guidelines on cardio-oncology developed in collaboration with the European Hematology Association (EHA), the European Society for Therapeutic Radiology and Oncology (ESTRO) and the International Cardio-Oncology Society (IC-OS). Eur Heart J 2022. |

1. **Statements: Pharmacology**

| **No** | **Area of competence** | **Statement** |
| --- | --- | --- |
| 1 | Treatment considerations for medical conditions in nursing (breastfeeding) women | Medication treatments (drug therapies) are sometimes withheld from breastfeeding women to prevent potentially harmful effects on the child. This results in women experiencing ongoing discomfort from symptoms and/or poor control of their own medical conditions.  We need clinicians to be aware of existing evidence that guides safe prescription for breastfeeding women with minimal or no actual risks to the child.  We need expanded research to understand the properties of existing and newly developed drugs in breast milk, including any relationships between breastfeeding and safer timing of doses taken by the woman. |
| 2 | Treatment considerations for medical conditions in pregnant women | Medication treatments (drug therapies) are sometimes withheld from pregnant women to prevent potential harm to the foetus. This results in women experiencing ongoing discomfort from symptoms and/or poor control of their own medical conditions. In some instances, withholding medication for the mother’s condition can actually lead to poor foetal outcomes. We need clinicians to be aware of existing evidence that guides safe prescription for pregnant women with minimal or no actual risks to the foetus. We need more research to understand how drugs affect foetal outcomes, especially the treatments for common conditions that affect women of childbearing age. |
| 3 | Treatment considerations for women who are intending to become pregnant | Medication treatments (drug therapies) are sometimes withheld from women of child-bearing potential (WOCBP) because the effect of the medicines on fertility are unknown and to prevent potentially harmful effects on the child. This approach results in women experiencing ongoing discomfort from symptoms and/or poor control of their own medical conditions.  On the other hand, certain potentially teratogenic medications are prescribed to WOCBP despite known risk to the child.  We need clinicians to be aware of existing evidence that guides safe prescription for women who intend to become pregnant with minimal or no actual risks to the woman’s fertility nor to the child. At the same time, we need to educate clinicians on medications that are known to have the potential to cause infertility or to be teratogenic and ensure that clinicians counsel patients regarding these effects.  We need expanded research to understand the properties of existing and newly developed drugs that might affect fertility and the foetus. |
| 4 | Treatment considerations for post-menopausal women: unnecessary tests and unapproved therapies (i.e. herbal) | Post-menopausal symptoms, such as vaginal dryness, sexual discomfort, urinary symptoms, hot flashes, night sweats, depression, insomnia, and hair loss, are sometimes not understood as being part of post-menopausal syndrome. Consequently, post-menopausal women often undergo unnecessary tests, and if they are not offered evidence-based treatment, they start self-medication with unapproved therapies (either medicines or herbal therapies). We need clinicians to understand the pathophysiology of post-menopausal symptoms and to be aware of existing evidence-based treatment options for these conditions. |

**Pharmacology References**

| **Statement** | **References** |
| --- | --- |
| 1 | - A comprehensive review on non-clinical methods to study transfer of medication into breast milk – A contribution from the ConcePTION project. Nauwelaerts N, Deferm N, Smits A, Bernardini C, Lammens B, Gandia P, Panchaud A, Nordeng H, Bacci LM, Forni M, Ventrella D, Van Calsteren K, DeLise A, Huys I, Bouisset-Leonard M, Allegaert K, Annaert P. Biomedicine & Pharmacotherapy. 2021;136: doi <https://doi.org/10.1016/j.biopha.2020.111038>. |
| 2 | - Where are the data linking infant outcomes, breastfeeding and medicine exposure? A systematic scoping review. Jordan S, Komninou S, Lopez Leon S (2023) PLoS ONE 18(4): e0284128. <https://doi.org/10.1371/journal.pone.0284128> |
| 3 | - Buchanan JF, Davis LJ. Drug-induced infertility. Drug Intell Clin Pharm. 1984 Feb;18(2):122-32. doi: 10.1177/106002808401800205. PMID: 6141923. - Pace LE, Schwarz EB. Balancing act: safe and evidence-based prescribing for women of reproductive age. Womens Health (Lond). 2012 Jul;8(4):415-25. doi: 10.2217/whe.12.25. PMID: 22757732. - Panchal BD, Cash R, Moreno C, Vrontos E, Bourne C, Palmer S, Simpson A, Panchal AR. High-Risk Medication Prescriptions in Primary Care for Women Without Documented Contraception. J Am Board Fam Med. 2019 Jul-Aug;32(4):474-480. doi: 10.3122/jabfm.2019.04.180281. PMID: 31300567. - Tajima K, Tsuchiya M, Ishikawa T, Obara T, Mano N. Real-world anticancer medications for reproductive-age women with breast cancer by using a claims database in Japan. Future Oncol. 2021 May;17(15):1907-1921. doi: 10.2217/fon-2020-1053. Epub 2021 Feb 24. PMID: 33625252. |
| 4 | - Dalal PK, Agarwal M. Postmenopausal syndrome. Indian J Psychiatry. 2015 Jul;57(Suppl 2):S222-32. doi: 10.4103/0019-5545.161483. PMID: 26330639; PMCID: PMC4539866. |

1. **Statements: Endocrinology**

| **No** | **Area of competence** | **Statement** |
| --- | --- | --- |
| 1 | Diabetes and obesity | The global prevalence of type 2 diabetes is increasing, with 437.9 million cases (5282.9 per 100,000) and 66.3 million DALYs in 2019. almost 60% of adults in Europe are either overweight or have obesity (add WHO 2022 as reference). Men are more often overweight at a younger age but women have a higher risk of obesity especially after menopause. Obesity in younger women is related to lower fertility rates as well as pregnancy complications. Psychosocial stress is a greater risk factor for obesity and type 2 diabetes in women than men. Women with obesity and/or diabetes feature greater relative risk of cardiovascular events and heart failure compared to men with obesity and/or diabetes.  In adult women, healthcare practitioners should screen for and treat diabetes, and other cardiovascular risk factors, assess overweight and obesity, and develop a weight management plan. |
| 2 | Hypercholesterolemia | Cardiovascular disease is the leading cause of death in women, with 8.9 million deaths in 2019, most due to atherosclerotic ischemic heart disease (e.g., MI or heart attack), which is directly related to high levels of low density lipoprotein cholesterol (LDL-C).  Meta-analyses of more than 20 randomised cardiovascular outcome trials show that reducing LDL-C by 1mmol/L with statins reduces the risk of cardiovascular events by 22% over 5 years of treatment. Yet, high cholesterol in women is underdiagnosed and undertreated, possibly because of misconceptions that statins do not benefit women, especially pre-menopausal women, and may be less safe than in men.  In women age 40 and older with borderline 10 year risk of cardiovascular disease statin treatment was cost saving, In women of this age group with even higher LDL-C statin treatment was highly cost effective (ICER $18,487 per QALY gained). Medical practitioners should screen for elevated cholesterol in women, assess cardiovascular disease risk, and use statins to reduce LDL-C and atherosclerotic cardiovascular disease. |
| 3 | Thyroid disease | Thyroid disease affects about 12% of women during their lifetime.  Deficiency of iodine which is essential for production of thyroid hormone is estimated to occur in 40% of the global population, and in 2010 contributed to 4 million disability adjusted life years lost. Thyroid disease is 10 times more common in women compared to men. This difference is likely related to sex differences in immune function, which lead to more autoimmune diseases in women, including thyroiditis, Hashimoto’s thyroiditis, and Graves’ disease.  Screening for hypothyroidism with TSH is important throughout the lifetime, and especially critical before pregnancy or in early pregnancy. Screening all pregnant women for autoimmune thyroid disease by TSH testing has been shown to be cost effective, with an estimated incremental cost-effectiveness ratio (ICER) of $4956/QALY compared to no screening, assuming that untreated hypothyroidism had no effect on IQ. Medical practitioners should strongly consider screening pregnant women with TSH to detect and treat hypothyroidism to avoid adverse outcomes for the mother, foetus, and child. |
| 4 | Osteoporosis | Osteoporosis in women, is twice as common compared to men, and estimated to occur in 23% of women and 11.7% of men. The most serious outcome of osteoporosis is bone fracture, which is associated with increased mortality.  Complications of hip fracture that may contribute to death are pulmonary embolism, infections and heart failure. Osteoporosis in women usually occurs with menopause and aging. In men, the diagnosis of osteoporosis indicates another disorder(e.g. Cushing, alcoholism).  To prevent osteoporosis and bone fracture, medical practitioners should counsel women on preventive measures such as a healthy diet with foods rich in calcium, limited consumption of alcohol, smoking cessation and physical activity (especially with weight-bearing exercises such as walking, jogging, and climbing stairs). |

**Endocrinology References**

| **Statement** | **References** |
| --- | --- |
| 1 | - Safiri S, Karamzad N, Kaufman JS, Bell AW, Nejadghaderi SA, Sullman MJM, Moradi-Lakeh M, Collins G, Kolahi AA. [**Prevalence**, **Deaths** and **Disability**-**Adjusted**-**Life**-**Years** (DALYs) Due to Type 2 **Diabetes** and Its Attributable Risk Factors in 204 Countries and Territories, 1990-2019: Results From the Global Burden of Disease Study 2019.](https://pubmed.ncbi.nlm.nih.gov/35282442/) Front Endocrinol (Lausanne). 2022 Feb 25;13:838027. doi: 10.3389/fendo.2022.838027. eCollection 2022. PMID: 35282442 - Global Burden of Disease Study 2019 (GBD 2019) Covariates 1980-2019 \| GHDx. [https://ghdx.healthdata.org/record/global-burden-disease-study-2019-gbd-2019-covariates-1980-2019 n.d](https://ghdx.healthdata.org/record/global-burden-disease-study-2019-gbd-2019-covariates-1980-2019%20n.d) - Boutari C and Mantzoros CS. A 2022 update on the epidemiology of obesity and a call to action: as its twin COVID-19 pandemic appears to be receding, the obesity and dysmetabolism pandemic continues to rage on. [Metabolism.](https://www.ncbi.nlm.nih.gov/pmc/articles/PMC9107388/) 2022 Aug; 133: 155217. Published online 2022 May 15. doi: [10.1016/j.metabol.2022.155217](https://doi.org/10.1016%2Fj.metabol.2022.155217). PMCID: PMC9107388  PMID: [35584732](https://pubmed.ncbi.nlm.nih.gov/35584732) - Kautzky-Willer A, Leutner M, Harreiter J. Sex differences in type 2 diabetes. Diabetologia 2023 Jun;66(6):986-1002. doi: 10.1007/s00125-023-05891-x. |
| 2 | - Vogel B, Acevedo M, Appelman A et al.  The Lancet women and cardiovascular disease Commission: reducing the global burden by 2030.  Lancet 2021;  397(10292):2385-2438. doi: 10.1016/S0140-6736(21)00684-X. Epub 2021 May 16. PMID: 34010613 Review. - Cholesterol Treatment Trialists’ (CTT) Collaboration; Baigent C, Blackwell L, Emberson J, Holland LE, Reith C, Bhala N, Peto R, Barnes EH, Keech A, Simes J, Collins R.  [Efficacy and safety of more intensive lowering of LDL cholesterol: a meta-analysis of data from 170,000 participants in 26 randomised trials.](https://pubmed.ncbi.nlm.nih.gov/21067804/)  Lancet. 2010 Nov 13;376(9753):1670-81. doi: 10.1016/S0140-6736(10)61350-5. Epub 2010 Nov 8.PMID: 21067804 - [Cholesterol Treatment Trialists' (CTT) Collaboration](https://pubmed.ncbi.nlm.nih.gov/?term=Cholesterol+Treatment+Trialists%27+%28CTT%29+Collaboration%5BCorporate+Author%5D); [Jordan Fulcher](https://pubmed.ncbi.nlm.nih.gov/?term=Fulcher+J&cauthor_id=25579834), [Rachel O'Connell](https://pubmed.ncbi.nlm.nih.gov/?term=O%27Connell+R&cauthor_id=25579834), [Merryn Voysey](https://pubmed.ncbi.nlm.nih.gov/?term=Voysey+M&cauthor_id=25579834), [Jonathan Emberson](https://pubmed.ncbi.nlm.nih.gov/?term=Emberson+J&cauthor_id=25579834), [Lisa Blackwell](https://pubmed.ncbi.nlm.nih.gov/?term=Blackwell+L&cauthor_id=25579834), [Borislava Mihaylova](https://pubmed.ncbi.nlm.nih.gov/?term=Mihaylova+B&cauthor_id=25579834), [John Simes](https://pubmed.ncbi.nlm.nih.gov/?term=Simes+J&cauthor_id=25579834), [Rory Collins](https://pubmed.ncbi.nlm.nih.gov/?term=Collins+R&cauthor_id=25579834), [Adrienne Kirby](https://pubmed.ncbi.nlm.nih.gov/?term=Kirby+A&cauthor_id=25579834), [Helen Colhoun](https://pubmed.ncbi.nlm.nih.gov/?term=Colhoun+H&cauthor_id=25579834), [Eugene Braunwald](https://pubmed.ncbi.nlm.nih.gov/?term=Braunwald+E&cauthor_id=25579834), [John La Rosa](https://pubmed.ncbi.nlm.nih.gov/?term=La+Rosa+J&cauthor_id=25579834), [T R Pedersen](https://pubmed.ncbi.nlm.nih.gov/?term=Pedersen+TR&cauthor_id=25579834), [Andrew Tonkin](https://pubmed.ncbi.nlm.nih.gov/?term=Tonkin+A&cauthor_id=25579834), [Barry Davis](https://pubmed.ncbi.nlm.nih.gov/?term=Davis+B&cauthor_id=25579834), [Peter Sleight](https://pubmed.ncbi.nlm.nih.gov/?term=Sleight+P&cauthor_id=25579834), [Maria Grazia Franzosi](https://pubmed.ncbi.nlm.nih.gov/?term=Franzosi+MG&cauthor_id=25579834), [Colin Baigent](https://pubmed.ncbi.nlm.nih.gov/?term=Baigent+C&cauthor_id=25579834), [Anthony Keech](https://pubmed.ncbi.nlm.nih.gov/?term=Keech+A&cauthor_id=25579834). Efficacy and safety of LDL-lowering therapy among men and women: meta-analysis of individual data from 174,000 participants in 27 randomised trials. Lancet. 2015 Apr 11;385(9976):1397-405.  PMID: **25579834.**  doi: 10.1016/S0140-6736(14)61368-4. Epub 2015 Jan 9. - Kohli-Lynch CN, Bellows BK, Thanassoulis G, Zhang Y, Pletcher MJ, Vittinghoff E, Pencina MJ, Kazi D, Sniderman AD, Moran AE.. [Cost-effectiveness of Low-density Lipoprotein Cholesterol Level-Guided Statin Treatment in Patients With Borderline Cardiovascular Risk.](https://pubmed.ncbi.nlm.nih.gov/31461121/) JAMA Cardiol. 2019 Oct 1;4(10):969-977. doi: 10.1001/jamacardio.2019.2851.PMID: 31461121 - Kohli-Lynch CN, Bellows BK, Zhang Y, Spring B, Kazi DS, Pletcher MJ, Vittinghoff E, Allen NB, Moran AE. Cost-effectiveness of lipid -lowering treatments in young adults. .J Am Coll Cardiol. 2021 Nov 16;78(20):1954-1964. doi: 10.1016/j.jacc.2021.08.065.PMID: 34763772 |
| 3 | - Mammen JSR, Coppola AR. Autoimmune thyroid disease in women.  JAMA. 2021 Jun 15;325(23):2392-2393. doi: 10.1001/jama.2020.22196.PMID: 33938930 - Dosiou C, Sanders GD, Araki SS, Crapo LM. Screening pregnant women for autoimmune thyroid disease: a cost-effectiveness analysis.  European J Endocrinol. 2008; 158: 841-851. |
| 4 | - Salari, N., Ghasemi, H., Mohammadi, L. et al. The global prevalence of osteoporosis in the world: a comprehensive systematic review and meta-analysis. J Orthop Surg Res 16, 609 (2021). https://doi.org/10.1186/s13018-021-02772-0 - Panula, J., Pihlajamäki, H., Mattila, V.M. et al. Mortality and cause of death in hip fracture patients aged 65 or older - a population-based study. BMC Musculoskelet Disord 12, 105 (2011). <https://doi.org/10.1186/1471-2474-12-105> |

1. **Statements: Neurology**

| **No** | **Area of competence** | **Statement** |
| --- | --- | --- |
| 1 | Migraine | Women with migraine do not always receive the treatment they need. The American Migraine Prevalence and Prevention (AMPP) study reported a cumulative incidence of lifetime migraine in 43% of women and 18% of men (1). Women suffer migraine more frequently than men do, and they also perceive the migraine as being more severe. For example, compared to men who suffer from migraine, women report more symptoms, more headache-related disability, and higher pain intensities overall (3,4,5). Less than 13% of patients with episodic migraine take preventative medicine, despite the fact that this medicine would benefit about 38% of them (4). Thus, all healthcare professionals need to be aware that episodic and chronic migraine is a treatable and manageable condition, and all women with migraine need to receive qualified care on time. |
| 2 | Cerebral vein thrombosis (CVT) | The statistics that are currently available indicate that CVT is uncommon. It may occur at any age and in both sexes, but it has a 3 to 1 female preponderance, mostly due to the use of oral contraceptives and, to a lesser extent, to pregnancy, post-partum period, hormone replacement therapy, and in vitro fertilisation. Early treatment can greatly improve the prognosis of CVT, save patients’ lives, improve their life quality, and reduce family and social burdens (1,2). Misdiagnosis and delayed diagnosis are frequent, with up to 73% of patients receiving an initial misdiagnosis and 40% experiencing diagnosis delays of more than 10 days (3). Healthcare professionals should be aware of these sex specific risk factors and consider risk factors specific to women. Assess patients with new-onset or altered headache especially carefully. |
| 3 | Treatment of epilepsy with valproic acid in women of childbearing age | In pregnant epileptic women, maternal safety and the possible teratogenicity of anticonvulsants must be carefully considered. According to recently accumulating research, valproic acid (VPA) may cause postnatal congenital abnormalities and impair cognitive function (1,2,3). For instance, in February 2018, the Pharmacovigilance Risk Assessment Committee of the European Medicines Agency recommended that women of childbearing age who are not enrolled in a pregnancy prevention program should not be prescribed valproate unless they have a form of epilepsy that is unresponsive to other anti-epileptic medications (4). An estimated 10.7% of children exposed to valproate in utero are born with a significant congenital deformity, i.e., a structural anomaly that is visible at birth, versus an estimated 2% of children who were not exposed to the drug (1). This difference in percentage indicates two issues: patients lack the expertise to weigh these risks, and clinicians frequently explain the risks poorly (5). All doctors who treat women with epilepsy in their fertile phase need to be aware of the risks of valproic acid and discuss them with their patients. |
| 4 | Women and stroke | Despite a lower incidence rate of stroke among women, women have a higher lifetime prevalence of stroke than men and develop an estimated 55 000 more strokes than men in the United States each year (1). The main and the most common widely known symptoms of brain stroke are well-known (hemisyndrome, incomprehensive speech, facial asymmetry), but women more often than men indicate non-specific and sometimes confusing symptoms, such as nausea, dizziness, weakness, headache, hiccups, chest pain, elevated heart rate, and confusion. These non-specific symptoms can mislead the doctor and the diagnosis of stroke will be delayed or missed all together (2,3). It is crucial that doctors at least are aware of the potential sex variations in stroke presentation, especially for non-focal symptoms such as headache and changes in mental state. This knowledge may be vital for women who experience sudden onset of non-focal symptoms but no clear focal signs of a stroke (3,4). |

**Neurology References**

| **Statement** | **References** |
| --- | --- |
| 1 | - Allais G, Chiarle G, Sinigaglia S et al (2018) Menstrual migraine: a review of current and developing pharmacotherapies for women. Expert Opin Pharmacother 19(2):123–136 - Burch R., Rizzoli P., Loder E.: The prevalence and impact of migraine and severe headache in the United States: figures and trends from government health studies. Headache 2018; 58: pp. 496-505. - Lipton R.B., Serrano D., Holland S., et. al.: Barriers to the diagnosis and treatment of migraine: effects of sex, income, and headache features. Headache 2013; 53: pp. 81-92. - Ha H, Gonzalez A. Migraine Headache Prophylaxis. Am Fam Physician. 2019 Jan 1;99(1):17-24. PMID: 30600979. - Allais G, Chiarle G, Sinigaglia S, Airola G, Schiapparelli P, Benedetto C. Gender-related differences in migraine. Neurol Sci. 2020 Dec;41(Suppl 2):429-436. doi: 10.1007/s10072-020-04643-8. PMID: 32845494; PMCID: PMC7704513. |
| 2 | - Coutinho JM, Ferro JM, Canhão P, Barinagarrementeria F, Cantú C, Bousser MG, Stam J. Cerebral venous and sinus thrombosis in women. Stroke. 2009 Jul;40(7):2356-61. doi: 10.1161/STROKEAHA.108.543884. Epub 2009 May 28. PMID: 19478226. - Ropper AH, Klein JP. Cerebral Venous Thrombosis. N Engl J Med. 2021 Jul 1;385(1):59-64. doi: 10.1056/NEJMra2106545. PMID: 34192432. - Shah CT, Rizqallah JJ, Oluwole O, Kalnins A, Sheagren JN. Delay in diagnosis of cerebral venous and sinus thrombosis: successful use of mechanical thrombectomy and thrombolysis. Case Rep Med. 2011;2011:815618. doi: 10.1155/2011/815618. Epub 2011 Jul 9. PMID: 21776277; PMCID: PMC3138110. |
| 3 | - Ito M, Kinjo T, Seki T, Horie J, Suzuki T. The long-term prognosis of hippocampal neurogenesis and behavioral changes of offspring from rats exposed to valproic acid during pregnancy. Neuropsychopharmacol Rep. 2021 Jun;41(2):260-264. doi: 10.1002/npr2.12181. Epub 2021 May 5. PMID: 33949804; PMCID: PMC8340817. - Kinjo T, Ito M, Seki T, Fukuhara T, Bolati K, Arai H, Suzuki T. Prenatal exposure to valproic acid is associated with altered neurocognitive function and neurogenesis in the dentate gyrus of male offspring rats. Brain Res. 2019 Nov 15;1723:146403. doi: 10.1016/j.brainres.2019.146403. Epub 2019 Aug 22. PMID: 31446017. - Mohammadkhani R, Ghahremani R, Salehi I, Safari S, Karimi SA, Zarei M. Impairment in social interaction and hippocampal long-term potentiation at perforant pathway-dentate gyrus synapses in a prenatal valproic acid-induced rat model of autism. Brain Commun. 2022 Sep 5;4(5):fcac221. doi: 10.1093/braincomms/fcac221. PMID: 36092302; PMCID: PMC9453432. - Macfarlane A, Greenhalgh T. Sodium valproate in pregnancy: what are the risks and should we use a shared decision-making approach? BMC Pregnancy Childbirth. 2018 Jun 1;18(1):200. doi: 10.1186/s12884-018-1842-x. PMID: 29859057; PMCID: PMC5984824. - Meador K, Reynolds MW, Crean S, Fahrbach K, Probst C. Pregnancy outcomes in women with epilepsy: a systematic review and meta-analysis of published pregnancy registries and cohorts. Epilepsy Res. 2008 Sep;81(1):1-13. doi: 10.1016/j.eplepsyres.2008.04.022. Epub 2008 Jun 18. PMID: 18565732; PMCID: PMC2660205. |
| 4 | - Benjamin EJ, Virani SS, Callaway CW, Chamberlain AM, Chang AR, Cheng S, Chiuve SE, Cushman M, Delling FN, Deo R, de Ferranti SD, Ferguson JF, Fornage M, Gillespie C, Isasi CR, Jiménez MC, Jordan LC, Judd SE, Lackland D, Lichtman JH, Lisabeth L, Liu S, Longenecker CT, Lutsey PL, Mackey JS, Matchar DB, Matsushita K, Mussolino ME, Nasir K, O'Flaherty M, Palaniappan LP, Pandey A, Pandey DK, Reeves MJ, Ritchey MD, Rodriguez CJ, Roth GA, Rosamond WD, Sampson UKA, Satou GM, Shah SH, Spartano NL, Tirschwell DL, Tsao CW, Voeks JH, Willey JZ, Wilkins JT, Wu JH, Alger HM, Wong SS, Muntner P; American Heart Association Council on Epidemiology and Prevention Statistics Committee and Stroke Statistics Subcommittee. Heart Disease and Stroke Statistics-2018 Update: A Report From the American Heart Association. Circulation. 2018 Mar 20;137(12):e67-e492. doi: 10.1161/CIR.0000000000000558. Epub 2018 Jan 31. Erratum in: Circulation. 2018 Mar 20;137(12 ):e493. PMID: 29386200. - Bruce SS, Merkler AE, Bassi M, Chen ML, Salehi Omran S, Navi BB, Kamel H. Differences in Diagnostic Evaluation in Women and Men After Acute Ischemic Stroke. J Am Heart Assoc. 2020 Mar 3;9(5):e015625. doi: 10.1161/JAHA.119.015625. Epub 2020 Feb 28. PMID: 32106749; PMCID: PMC7335545. - Ali M, van Os HJA, van der Weerd N, Schoones JW, Heymans MW, Kruyt ND, Visser MC, Wermer MJH. Sex Differences in Presentation of Stroke: A Systematic Review and Meta-Analysis. Stroke. 2022 Feb;53(2):345-354. doi: 10.1161/STROKEAHA.120.034040. Epub 2021 Dec 14. PMID: 34903037; PMCID: PMC8785516. - Medlin F, Amiguet M, Eskandari A, Michel P. Sex differences in acute ischaemic stroke patients: clinical presentation, causes and outcomes. Eur J Neurol. 2020 Aug;27(8):1680-1688. doi: 10.1111/ene.14299. Epub 2020 Jun 8. PMID: 32383518. |

1. **Statements: Emergency Medicine**

| **No** | **Area of competence** | **Statement** |
| --- | --- | --- |
| 1 | Acute cardiovascular disease | Although there has been an increase in knowledge about the differences between women and men in terms of etiology, treatment and outcomes of acute cardiovascular disease, women still remain underdiagnosed and undertreated. A major barrier continues to be the persisting assumption that acute cardiovascular disease occurs primarily in men. There needs to be greater awareness regarding the potential differences between the differential diagnoses of acute cardiovascular disease in men and women. It is necessary to address sex-specific risk factors, symptomatology, and pathophysiology to prevent misdiagnosis, to ensure proper treatment and to reduce morbidity and mortality in women. The differential diagnoses, risk factors and treatment should be systematically taught at every stage of healthcare professional training. |
| 2 | Unconscious bias and misdiagnosis in cardiovascular disease | Sex and gender differences in all aspects of cardiovascular diseases have been known for decades. However, women are still more likely to be misdiagnosed and undertreated, which is to a large extent due to the unconscious bias that healthcare workers derived from the biased outdated educational materials which are still being used in healthcare education. It has been estimated that women are 50% more likely to be misdiagnosed than men. Misdiagnosis of a heart attack could increase the risk of death at 30 days after symptom onset by 70%. The unconscious bias relating to women and acute cardiovascular disease should be actively addressed throughout medical school and training, with consideration for each part of the patient pathway: pre-hospital, emergency medicine, in-patient, critical care, follow-up and outpatient, as well as relating to clinical research. |
| 3 | Symptomatology bias | Symptoms of acute cardiovascular and neurovascular disease in women should be taught, not as ‘atypical’ but rather as normal for women's health. It is rather curious that symptomatology in women is considered atypical, since women represent half our population. Current-day knowledge is often based on older literature and practice, which was male dominant. We need to change this bias in medical education to achieve equality in healthcare and not to consider sex differences as atypical. |
| 4 | The role of oestrogen | Oestrogen has important effects on vascular physiology and pathophysiology, which in turn has therapeutic implications. Oestrogen affects the vascular wall, the molecular mechanisms of vascular responsiveness, and atherosclerosis. Throughout a woman’s lifespan, oestrogen levels vary due to endogenous alterations as well as due to exogenous supplementation. These variations impact the incidence of acute cardiovascular and neurovascular diseases, as well as other important biological processes, such as drug metabolism. The impact of oestrogen on pharmacotherapy should be systematically embedded into guidelines, education and prescription recommendations regarding acute cardiovascular and neurovascular diseases. |
| 5 | Addressing sex differences in medical training | The training of future physicians and medical educators must evolve, supporting the promulgation of the most recent evidence-based understanding of the role of sex and gender in the diagnosis, treatment, and preventive care of our patients. There still is a lack of evidence and knowledge in many domains of women’s health. Therefore, basic science and translational research must address the effects of sex differences and sex hormones on disease processes, on the response to acute cardiovascular disease, on critical illness, and on recovery. This focus on sex differences should become a specific point of consideration in medical training and in  assessing grant proposals. |

**Emergency Medicine References**

| **Statement** | **References** |
| --- | --- |
| 1 | - Elsevier. (2020, 1 december). How Sex and Gender Impact Clinical Practice - 1st Edition. <https://www.elsevier.com/books/how-sex-and-gender-impact-clinical-practice/jenkins/978-0-12-816569-0>. - McGregor AJ, Beauchamp GA, Wira CR 3rd, Perman SM, Safdar B. Sex as a Biological Variable in Emergency Medicine Research and Clinical Practice: A Brief Narrative Review. West J Emerg Med. 2017 Oct;18(6):1079-1090. doi: 10.5811/westjem.2017.8.34997. Epub 2017 Oct 6. PMID: 29085541; PMCID: PMC5654878. - Keteepe-Arachi T, Sharma S. Cardiovascular Disease in Women: Understanding Symptoms and Risk Factors. Eur Cardiol. 2017 Aug;12(1):10-13. doi: 10.15420/ecr.2016:32:1. PMID: 30416543; PMCID: PMC6206467. |
| 2 | - Den Ruijter H. Sex and Gender Matters to the Heart. Front Cardiovasc Med. 2020 Nov 26;7:587888. doi: 10.3389/fcvm.2020.587888. PMID: 33330649; PMCID: PMC7732542. - Daugherty SL, Blair IV, Havranek EP, Furniss A, Dickinson LM, Karimkhani E, Main DS, Masoudi FA. Implicit Gender Bias and the Use of Cardiovascular Tests Among Cardiologists. J Am Heart Assoc. 2017 Nov 29;6(12):e006872. doi: 10.1161/JAHA.117.006872. PMID: 29187391; PMCID: PMC5779009. - ESC Scientific Document Group. 2021 ESC Guidelines for the diagnosis and treatment of acute and chronic heart failure: McDonagh TA, Metra M, Adamo M, Gardner RS, Baumbach A, Böhm M, Burri H, Butler J, Čelutkienė J, Chioncel O, Cleland JGF, Coats AJS, Crespo-Leiro MG, Farmakis D, Gilard M, Heymans S, Hoes AW, Jaarsma T, Jankowska EA, Lainscak M, Lam CSP, Lyon AR, McMurray JJV, Mebazaa A, Mindham R, Muneretto C, Francesco Piepoli M, Price S, Rosano GMC, Ruschitzka F, Kathrine Skibelund A;. Eur Heart J. 2021 Sep 21;42(36):3599-3726. doi: 10.1093/eurheartj/ehab368. Erratum in: Eur Heart J. 2021 Oct 14;: PMID: 34447992. |
| 3 | - McGregor AJ, Beauchamp GA, Wira CR 3rd, Perman SM, Safdar B. Sex as a Biological Variable in Emergency Medicine Research and Clinical Practice: A Brief Narrative Review. West J Emerg Med. 2017 Oct;18(6):1079-1090. doi: 10.5811/westjem.2017.8.34997. Epub 2017 Oct 6. PMID: 29085541; PMCID: PMC5654878. |
| 4 | - Miller VM, Duckles SP. Vascular actions of oestrogens: functional implications. Pharmacol Rev. 2008 Jun;60(2):210-41. doi: 10.1124/pr.107.08002. Epub 2008 Jun 25. PMID: 18579753; PMCID: PMC2637768. - Davezac M, Buscato M, Zahreddine R, Lacolley P, Henrion D, Lenfant F, Arnal JF, Fontaine C. Oestrogen Receptor and Vascular Aging. Front Aging. 2021 Sep 24;2:727380. doi: 10.3389/fragi.2021.727380. PMID: 35821994; PMCID: PMC9261451. |
| 5 | - McGregor AJ. The Need for Sex and Gender Education Reform. J Womens Health (Larchmt). 2015 Dec;24(12):961-2. doi: 10.1089/jwh.2015.5555. Epub 2015 Oct 8. PMID: 26447837; PMCID: PMC4683549. - McGregor AJ, Becker B. Food and Drug Administration Advances Drug Safety for Women: First Steps in a Long Journey. J Womens Health (Larchmt). 2021 Jul;30(7):911-913. doi: 10.1089/jwh.2020.8962. Epub 2021 Feb 12. PMID: 33577383. |

1. **Statements: Public Health**

| **No** | **Area of competence** | **Statement** |
| --- | --- | --- |
| 1 | Socioeconomic inequalities and women's health | Socioeconomic inequalities are well-known determinants of health outcomes. Due to complex social processes, socioeconomic inequalities often have a more severe impact on the health of women and girls than on the health of men and boys with whom they live. We highlight four pathways:   - when food is scarce, women and girls tend to eat less and less nutritious food than men and boys, so that the health of females generally suffers more during periods of food insecurity - in societies in which education is not fully accessible to all children from all families, families who have to compromise tend to let boys follow more education than girls - less education is associated with worse health outcomes, implying that the health of girls and women will be more affected than the health of boys and men in such situations of inequality; - pay-gaps between men and women are widespread - if low-status jobs are perceived as ‘female’ jobs, their wages are even lower than those for low-status ‘male’ jobs, due to childbirth and their gendered role as main carer, women typically spend fewer years in formal paid employment than men, because of tendencies such as these, women in disadvantaged socioeconomic conditions tend to have less income and pension than their male counterparts, in general, they are less likely to have access to household money. Less income is associated with worse health outcomes; - due to factors such as less education, less access to money/less income and gendered roles, women with low socioeconomic status tend to have less access to healthcare than men in similar positions.   Because socioeconomic inequalities tend to affect women’s overall health and well-being even more strongly than they affect men’s health, addressing socioeconomic inequalities is an essential step in promoting women’s health. Physicians can support their individual patients by systematically inquiring whether poverty might play a role in their choices for themselves and their family members, and by referring to support options. They can support girls and women in general by consistently and vividly sharing their stories about gendered inequality with politician, policy makers and the media. |
| 2 | Violence against women | There are many forms of violence against women: physical and sexual violence, female genital mutilation (FGM), psychological abuse, economic abuse, forced marriage, and human trafficking. Violence against women is often linked to gender inequality and discrimination, an issue affecting women and girls across all aspects of society. According to WHO estimates, more than 30% of women worldwide have experienced either physical or sexual partner violence, whereas 7% of women worldwide have experienced non-partner sexual assault. Worldwide, approximately 100–140 million girls and women have undergone female genital mutilation (FGM) and many girls are still at risk.  Violence against women may severely impact the victim’s health. Epidemiological and clinical studies show that intimate partner violence, for instance, is consistently associated with negative physical and mental health outcomes, such as a higher incidence of gynaecological disorders, adverse pregnancy outcomes, irritable bowel syndrome, gastrointestinal disorders, various chronic-pain syndromes, depression, anxiety, phobias, post-traumatic stress disorder, suicidality, and alcohol and drug abuse.  Due to the high prevalence of violence against women and its severe adverse effects on both physical and mental health, it is absolutely imperative that physicians:   1. are aware of the magnitude of the problem and the severity of its impact 2. actively take part in prevention programmes for female genital mutilation 3. receive training in recognising symptoms and signs, and in tactfully and effectively addressing them during consultations.   Refer patients in an effective and safe manner to support programs and legal aid. |
| 3 | Reproductive and well-childcare services as central to improving the health of women – and of the population they are part of | The improvement of women’s reproductive health is a central concern in the UN’s Sustainable Development Goal (SDG) 3, to ensure healthy lives and well-being for all. The UN has set targets for reduction of the maternal mortality ratio and of neonatal and early child mortality, which are still far from realised. The vaccination rate of children has even declined during the Covid-19 pandemic. Furthermore, the UN aims to have realised by 2030 ‘universal access to sexual and reproductive health-care services, including for family planning, information and education, and the integration of reproductive health into national strategies and programmes’. Important steps have been taken. For instance, by 2023 57.4% of women of reproductive age (15-49 years) in Sub-Saharan Africa have their need for family planning satisfied with modern contraceptive methods, an increase of almost 6% since 2015. Yet, universal and worldwide access to these services seems out of reach. In some developed countries such as the United States, access to reproductive education and to abortion has recently been declining.  Physicians should be aware of the crucial importance of reproductive healthcare and well-childcare services in order to achieve health and well-being for women, their children, and ultimately for the whole population. In order to ensure that women have access to a broad range of high-quality, comprehensive reproductive and well-childcare services, physicians can:   - promote medical education and research funding on the care and themes covered by reproductive and well-childcare services; - support their colleagues working for these services; ensure that they are backed up by adequate resources and staff; take care that cooperation with these colleagues is smooth and open; - speak up in favour of reproductive services, and vehemently fight back in case of any attack on aspects of reproductive services. |
| 4 | Community participation in the development of reproductive health | Reproductive health programmes and services are central in supporting and sustaining women’s health. Yet, in order to successfully realise their goals, such programmes need to ensure community participation. This process can be compared to shared-decision making in individual healthcare. Just as individual patients can best assess their goals and needs, and adapt their care path to them, community members themselves know best how community services ought to be designed for maximal response, connection and effectiveness. A representative group of community members should therefore be involved in needs assessments, providing input on programme design and implementation, and helping to identify and address local reproductive health challenges and issues. The effectiveness of the programmes can be greatly enhanced, if they also participate in planning, implementing and evaluating the reproductive health programmes and services. Key persons from the community can greatly advance programs if they can meaningfully support them as ambassador. Since community participation is essential and valuable work, participants should be adequately rewarded for it.  With regard to this theme, physicians should be aware of the need for community participation when designing, implementing and evaluating reproductive programmes and services, parallel to shared-decision making in individual healthcare. They should know that many tried-and-tested techniques support meaningful community participation, such as Participatory Action Research, human-centred design, Photo Voice, narrative techniques and citizen’s council. Backed up by this knowledge, they should actively promote community participation from the earliest stages of setting up reproductive programmes and services. |
| 5 | The importance of screening in the prevention of malignant diseases in women | Screening is essential to preventing malignant diseases (cancers) in women. Several types of screening tests can be used to detect malignant diseases in women, including breast cancer screening, cervical cancer screening, colorectal cancer screening, etc. Cervical cancer for instance is responsible for more than 7% of all cancer-related deaths in women worldwide. Nowadays, the large majority of cases of cervical cancer (85%) occur in developing countries that have ineffective screening programmes. Its annual incidence and mortality rate has significantly declined in developed countries that implemented screening; the United States report a decrease of 50% since 1975.  Next to inequity in screening between developed and developing countries, studies point to inequity for access to screening programmes within developed countries as well. Women with unstable housing conditions, with drug use or with severe psychiatric illnesses all tend to have higher risk for different malignant diseases. Yet, non-compliance to screening programs tends to be significantly higher among these groups, leading to worse health outcomes.  Physicians should be aware of the inequity in compliance with and outcomes of cancer screening programs for women. They should promote the compliance with screening programs amongst all women. They should be aware of the challenges faced by women from disadvantaged groups in complying with standard screening programmes. Physicians can tactfully yet actively support these women to adhere to screening programs, with potentially substantial beneficial effects for their health. |

**Public Health References**

| **Statement** | **References** |
| --- | --- |
| 1 | - Global Strategy for Women’s, Children’s and Adolescents’ Health (2016-2030). Available online: https://www.everywomaneverychild.org/wp-content/uploads/2017/10/EWEC_GSUpdate_Brochure_EN_2017_web.pdf [accessed on 25/01/2023]. - WHO, CSDH. Closing the gap in a generation: health equity through action on the social determinants of health. Geneve: WHO 2008. Available online: [accessed on 25/01/2023]. - Vogel B, et al. The Lancet women and cardiovascular disease Commission: reducing the global burden by 2030. Lancet 2021; DOI: https://doi.org/10.1016/S0140-6736(21)00684-X - Langer A, et al. Women and Health: the key for sustainable development. Lancet 2015; 386: 1165–210. DOI: http://dx.doi.org/10.1016/S0140-6736(15)60497-4 - Moss NE. Gender equity and socioeconomic inequality: a framework for the patterning of women's health. Social Science & Medicine 2002; 54(5): 649-661. DOI: https://doi.org/10.1016/S0277-9536(01)00115-0. - Quizhpe E, Sebastian MS, Teran E, et al. Socioeconomic inequalities in women’s access to health care: has Ecuadorian health reform been successful? Int J Equity Health 2020; 19: 178. DOI: https://doi.org/10.1186/s12939-020-01294-1 - Manandhar, M.; Hawkes, S.; Buse, K.; Nosrati, E.; Magar, V. Gender, health and the 2030 agenda for sustainable development. Bull World Health Organ. 2018, 96[9]:644–653. DOI:10.2471/BLT.18.211607. - Mishra P.K., Mishra S.K., Sarangi M.K. Do Women’s Advancement and Gender Parity Promote Economic Growth? Evidence from 30 Asian Countries. Millennial Asia 2020; 11[1: 5–26. DOI: https://doi.org/10.1177/0976399619900603 - UN General Assembly. Transforming our world: the 2030 Agenda for Sustainable Development. United Nations: New York, USA, 2017. Available online: https://www.un.org/en/development/desa/population/migration/generalassembly/docs/globalcompact/A_RES_70_1_E.pdf [accessed on 25/01/2023]. - Shannon G., Jansen M., Williams K., Cáceres C., Motta A., Odhiambo A., Eleveld A., Mannell J. Gender equality in science, medicine, and global health: where are we at and why does it matter? Lancet 2019; 393: 560–69. DOI: https://doi.org/10.1016/S0140-6736[18]33135-0 [accessed on 25/01/2023]. - World Economic Forum [WEF]. Global gender gap report 2020. Geneva: The World Economic Forum. Available online: https://reports.weforum.org/global-gender-gap-report-2020/ [accessed on 25/01/2023]. |
| 2 | - Ellsberg, et al. Prevention of violence against women and girls: what does the evidence say? Lancet 2014; DOI: <http://dx.doi.org/10.1016/S0140-6736(14)61703-7> - Ellsberg, et al. Intimate partner violence and women's physical and mental health in the who multi-country study on women's health and domestic violence: an observational study. Lancet 2018; DOI: <https://doi.org/10.1016/S0140-6736(08)60522-X>. - Stark L, Robinson MV, Seff I, Gillespie A, Colarelli J, Landis D. The Effectiveness of Women and Girls Safe Spaces: A Systematic Review of Evidence to Address Violence Against Women and Girls in Humanitarian Contexts. Trauma, Violence, & Abuse 2022; 23(4): 1249–1261. DOI: https://doi.org/10.1177/1524838021991306 - WHO. Responding to intimate partner violence and sexual violence against women. WHO clinical and policy guidelines. Geneva: WHO Press 2013. |
| 3 | - United Nations, Sustainable Development, SDG 3: Ensure healthy lives and promote well-being for all at all ages. [Goal 3 \| Department of Economic and Social Affairs (un.org)](https://sdgs.un.org/goals/goal3). - WHO, GHWN, WGH. Delivered by women, led by men: A gender and equity analysis of the global health and social workforce. Human Resources for Health, Observer Series, 2019; 24. Available online: https://apps.who.int/iris/handle/10665/311322 [accessed on 09/01/2023]. - WHO. Global strategy on human resources for health Workforce. WHO: Geneva, Switzerland, 2030. Available online: https://apps.who.int/iris/bitstream/handle/10665/250368/9789241511131-eng.pdf [accessed on 09/01/2023]. - Gupta N., Balcom S.A., Singh P. Looking beyond parity: Gender wage gaps and the leadership labyrinth in the Canadian healthcare management workforce. Healthcare Management Forum 2023; 36[1]: 49–54. DOI: 10.1177/08404704221104435. - WHO. Geneva: Department of Reproductive Health and Research 2010. Available online: https://apps.who.int/iris/bitstream/handle/10665/70501/WHO?sequence=1 [accessed on 09/01/2023]. |
| 4 | - WHO Europe. Building resilience: a key pillar of Health 2020 and the Sustainable Development Goals. Examples from the WHO Small Countries Initiative. Copenhagen: Regional Office for Europe; 2017. - Center for Community Health and Development. The Community Tool Box: A Web-Based Resource for Building Healthier Communities. Lawrence, KS: The University of Kansas; 2019. https://ctb.ku.edu/en [accessed on 25/01/2023]. - Silumbwe A, et al. Facilitating community participation in family planning and contraceptive services provision and uptake: community and health provider perspectives. Reprod Health 12020; 7: 119. DOI: <https://doi.org/10.1186/s12978-020-00968-x> |
| 5 | - Siegel RL, Miller KD, Jemal A. Cancer statistics, 2018. CA Cancer J Clin 2018; 68: 7–30. DOI: 10.3322/caac.21442. - Rerucha CM, Caro RJ, Wheeler VL. Cervical Cancer Screening. Am Fam Physician 2018; 97(7): 441-448. Available online: https://www.aafp.org/dam/brand/aafp/pubs/afp/issues/2018/0401/p441.pdf. [accessed on 09/01/2023]. - Winters S, Martin C, Murphy D, Shokar NK. Chapter One - Breast Cancer Epidemiology, Prevention, and Screening, In: Lakshmanaswamy R, ed. Progress in Molecular Biology and Translational Science 2017; 151: 1-32. DOI: https://doi.org/10.1016/bs.pmbts.2017.07.002. - Loomans-Kropp HA, Umar A. Cancer prevention and screening: the next step in the era of precision medicine. NPJ Precis Oncol 2019; 28; 3:3. DOI: 10.1038/s41698-018-0075-9. - Garpenhag L, Dahlman D. Participation in screening for breast and cervical cancer among women with current or previous drug use: a survey study. BMC Public Health. 2023 Feb 16;23(1):352. doi: 10.1186/s12889-023-15236-3. PMID: 36797737; PMCID: PMC9936631. |

1. **Statements: Oncology**

| **No** | **Area of competence** | **Statement** |
| --- | --- | --- |
| 1 | Understanding the specificities of non-sex-related cancer in women (such as lung cancer) | There are gender-specific standards for the management of cancers that directly affect organs related to reproduction, such as ovarian and breast cancer in women and prostate cancer in men. Other cancers are considered not to be related to hormones and are treated regardless of gender. However, several translational studies have demonstrated that solid tumours, including gastric cancer and lung cancer, can be partially dependent on hormonal factors that impact therapeutic efficacy and toxicity. For example, the expression of oestrogen receptors is commonly reported in tumoral lung tissue. Hormonal replacement therapy is associated with a reduced risk of lung cancer in women but also with a decreased survival in women who already have lung cancer. Healthcare practitioners should consider these particularities to fine-tune therapeutic strategies in women. |
| 2 | Recognition of cancer treatment specificities (both efficacy and toxicity) in women | Solid tumours are treated with surgery, radiotherapy, chemotherapy, immunotherapy, or targeted therapy, depending on tumour stage and molecular background. It is well reported that the sensitivity to those treatments may vary between men and women. Nevertheless, dosage for immunotherapy and targeted therapy is exactly the same for all patients, regardless of gender, comorbidities and weight, which can lead to an increase in toxicity in women. Moreover, studies involving animal models and clinical trials to identify the appropriate dosage of drugs have almost all been male-oriented.  Medical practitioners should specifically study the pharmacokinetics of anti-cancer agents in female patients in preclinical and clinical studies to optimise the safety and efficacy of cancer treatment in women. |
| 3 | Favouring preclinical and translational research to address the specificities of cancer in women (outside breast and gynaecologists cancers) | The molecular background of cancer differs between male and female patients. For example, lung cancer in women is characterised by a higher proportion of somatic genetic alterations, including EGFR (25% vs 10%) or HER2 mutations. Due to these alterations, treatment based on oral targeted therapies show better outcomes than chemotherapy. A better knowledge of genetic alterations driving cancer in women will lead to more efficient treatment and better outcomes. Moreover, gender-oriented clinical trials focussing on women with cancer harbouring specific alterations will help to improve personalised cancer treatment. |
| 4 | Specifically addressing the bias in the interpretation of symptoms in women in the field of oncology | Many symptoms are interpreted differently in men and in women. For instance, pain, cough, neurological impairment, and gastro-intestinal symptoms are more often considered “functional” or “psychogenic” in women than in men. Moreover, some cancers, such as head and neck cancers, lung cancers, and GI cancers, are considered to be “male” diseases, which leads to a delay in both the diagnosis and the management of these cancers in women. We also observed that women are less frequently targeted by certain cancer screening programmes. However, the epidemiology of cancer is changing. For example, in many western countries, lung cancer is now as frequent in females as in males.  Medical practitioners and the general population should be better informed about the epidemiology of cancer to prevent under-consideration of cancer in women. |

**Oncology References**

| **Statement** | **References** |
| --- | --- |
| 1 | - [Characteristics of lung cancer in women: importance of hormonal and growth factors.](https://pubmed.ncbi.nlm.nih.gov/22197614/) Rouquette I, Mazières J. Lung Cancer. 2012 Jun;76(3):280-5. - [Hormone replacement therapy is associated with decreased survival in women with lung cancer.](https://pubmed.ncbi.nlm.nih.gov/16314616/) Ganti AK, et al. J Clin Oncol. 2006 Jan 1;24(1):59-63. - [Hormone replacement therapy and lung cancer risk in women: a meta-analysis of cohort studies: Hormone replacement therapy and lung cancer risk.](https://pubmed.ncbi.nlm.nih.gov/31860945/) Jin C, et al. Medicine (Baltimore). 2019. |
| 2 | - Wang, J. and Huang, Y. (2007) Pharmacogenomics of sex difference in chemotherapeutic toxicity. Curr. Drug Discov. Technol. 4, 59-68. - Tran, C., Knowles, S. R., Liu, B. A. and Shear, N. H. (1998) Gender differences in adverse drug reactions. J. Clin. Pharmacol. 38, 1003-1009. - Zucker, I. and Beery, A. K. (2010) Males still dominate animal studies. Nature 465, 690. |
| 3 | - [Routine molecular profiling of patients with advanced non-small-cell lung cancer: results of a 1-year nationwide programme of the French Cooperative Thoracic Intergroup (IFCT).](https://pubmed.ncbi.nlm.nih.gov/26777916/) - Barlesi F, Mazieres J, et al .Lancet. 2016 Apr 2;387(10026):1415-1426. - [Randomized Phase II Trial Evaluating Treatment with EGFR-TKI Associated with Antiestrogen in Women with Nonsquamous Advanced-Stage NSCLC: IFCT-1003 LADIE Trial.](https://pubmed.ncbi.nlm.nih.gov/32144133/) - Mazieres J, Barlesi F, et al Clin Cancer Res. 2020 Jul 1;26(13):3172-3181. |
| 4 | - [Reduced lung-cancer mortality with low-dose computed tomographic screening.](https://pubmed.ncbi.nlm.nih.gov/21714641/) - National Lung Screening Trial Research Team; Aberle DR, Adams AM, Berg CD, Black WC, Clapp JD, Fagerstrom RM, Gareen IF, Gatsonis C, Marcus PM, Sicks JD. N Engl J Med. 2011 Aug 4;365(5):395-409. - Sex Differences in Cancer: Epidemiology, Genetics and Therapy - Hae-In Kim, Hyesol Lim and Aree Moon. Biomol Ther 2018 |

1. **Statements: Patient Advisory Board**

| **No** | **Area of competence** | **Statement** |
| --- | --- | --- |
| 1 | Awareness and accompaniment of women to adapt to changes in the body due to disease, treatment, and also hormones | In periods of illness, women’s bodies can change, due to the disease itself, the treatment, surgery and hormones. This change is often not discussed with patients and can come as surprise (1). Women are affected by change in their appearance, for example after breast cancer (2, 3). Changes to an individual’s body resulting from illness and treatment can have a substantial impact on their everyday life, particularly in relation to their body image (4). Women are more likely to experience body image issues than men, which is partially due to sociological concepts (5). There is a need for nuanced understanding of the complex inter-relationship between body image and clinical, demographic, functional, and psychosocial variables for women (4). Doctors should know about the impact of bodily changes on women and arrange guidance or mental support in preparing women for these changes. |
| 2 | Psychological support for disease, importantly following treatment when there is a need to adjust to a different body image (physically, emotionally, and regarding family bonds) | Going through illness not only affects the involved tractus, but also patients’ lives and mental status. They can experience what is called biographical disruption: this describes illness as a fundamental rupture in the fabric of everyday life (1). There are differences in coping mechanisms between men and women (2). Women benefit more from social and mental support during and after a period of severe illness, such as breast cancer (3). For many women, the period after the illness itself is still part of the illness, as they have to readjust to a different body image, recover physically and emotionally and readjust the family bonds. Doctors should know about this difference in coping mechanisms and inform patients as well as arranging psychological support at the start of a period of severe illness and continuing this support after treatment in the hospital has ended. Medical schools should teach about integrative care for women’s health. |
| 3 | Awareness and accompaniment of fertility preservation during treatment, both for breast cancer and other diseases | While cancer treatment (or another treatment that influences fertility) is being planned, all women should be informed about the possible side-effects of the treatment on sexuality, fertility, and body image. When it comes to fertility, it is important to inform the patient about expected outcomes for fertility and options for fertility preservation (1,2). Since the field of fertility preservation is relatively young and still in development, up-to-date guidelines should be consulted (2). All doctors should know if a treatment impairs fertility and if so, they should inform the patient and consult guidelines for the most up-to-date information and fertility preservation options. Medical schools should provide training about fertility preservation as a possible complement to treatment. |

**Patient Advisory Board References**

| **Statement** | **References** |
| --- | --- |
| 1 | - Focus group meeting patient board - Davis C, Tami P, Ramsay D, Melanson L, MacLean L, Nersesian S, Ramjeesingh R. Body image in older breast cancer survivors: A systematic review. Psychooncology. 2020 May;29(5):823-832. doi: 10.1002/pon.5359. Epub 2020 Feb 20. PMID: 32048373. - Helms RL, O'Hea EL, Corso M. Body image issues in women with breast cancer. Psychol Health Med. 2008 May;13(3):313-25. doi: 10.1080/13548500701405509. PMID: 18569899. - Dunne S, Fitch M, Semple C. Editorial: Body image following cancer treatment. Front Psychol. 2022 Nov 7;13:1068977. doi: 10.3389/fpsyg.2022.1068977. PMID: 36420400; PMCID: PMC9677346. - Quittkat HL, Hartmann AS, Düsing R, Buhlmann U, Vocks S. Body Dissatisfaction, Importance of Appearance, and Body Appreciation in Men and Women Over the Lifespan. Front Psychiatry. 2019 Dec 17;10:864. doi: 10.3389/fpsyt.2019.00864. PMID: 31920737; PMCID: PMC6928134. |
| 2 | - Bury M. Chronic illness as biographical disruption. Sociol Health Illn. 1982 Jul;4(2):167-82. doi: 10.1111/1467-9566.ep11339939. PMID: 10260456. - Li HJ, Sun JZ, Zhang QL, Wei DT, Li WF, Jackson T, Hitchman G, Qiu J. Neuroanatomical differences between men and women in help-seeking and coping strategy. Sci Rep. 2014 Jul 16;4:5700. doi: 10.1038/srep05700. PMID: 25027617; PMCID: PMC4099976. - Greenlee H, Balneaves LG, Carlson LE, Cohen M, Deng G, Hershman D, Mumber M, Perlmutter J, Seely D, Sen A, Zick SM, Tripathy D; Society for Integrative Oncology. Clinical practice guidelines on the use of integrative therapies as supportive care in patients treated for breast cancer. J Natl Cancer Inst Monogr. 2014 Nov;2014(50):346-58. doi: 10.1093/jncimonographs/lgu041. Erratum in: J Natl Cancer Inst Monogr. 2015 May;2015(51):98. PMID: 25749602; PMCID: PMC4411539. |
| 3 | - Miaja M, Platas A, Martinez-Cannon BA. Psychological Impact of Alterations in Sexuality, Fertility, and Body Image in Young Breast Cancer Patients and Their Partners. Rev Invest Clin. 2017 Jul-Aug;69(4):204-209. doi: 10.24875/ric.17002279. PMID: 28776605. - Donnez J, Dolmans MM. Fertility preservation in men and women: Where are we in 2021? Are we rising to the challenge? Fertil Steril. 2021 May;115(5):1089-1090. doi: 10.1016/j.fertnstert.2021.03.028. Epub 2021 Apr 3. PMID: 33823991. |

1. **Round 2 Delphi: additional statements**

| **No** | **Area of competence** | **Statement** |
| --- | --- | --- |
| 1 | Sleep Insomnia | One in four women has insomnia symptoms. Insomnia is more common in women, especially older women, than in men, this is due to unique hormonal changes. These include hormonal changes during menstruation, pregnancy and especially menopause. Over time, insomnia may increase risks for accidents (such as falls, car accidents, etc) and health problems, including diabetes and high blood pressure. Insomnia can be a symptom or side effect of another health problem or stand on its own. Proper treatment is important: treating the underlying health problem, lifestyle advice (including sleep/wake schedule), cognitive behavioral therapy and prescriptions. Every physician working with women should have foundational knowledge about insomnia, since this is such a common problem in women and accurate treatment is essential, also in prevention of secondary health problems resulting from insomnia. |
| 2 | Auto-immune disease in women | Approximately 80% of all patients diagnosed with autoimmune diseases are women. Women tend to develop autoimmune diseases more often than men throughout the course of their lifetime. This could be due to the association of autoimmune conditions with the X chromosome. Women normally have two X chromosomes, and for this reason, possess a higher risk of autoimmune diseases, as compared to men. Auto-immune diseases know phases of remission and flares and can be hard to recognize, since symptoms can be general. Over 40% of women eventually diagnosed with auto-immune disease, have first been told that there is no underlying condition. Since this auto-immune disease are easily misdiagnosed or under recognized, physicians working with women should have foundational knowledge of symptoms in auto-immune disease, so they can properly refer women for accurate diagnosis. |
| 3 | Peri-operative care in women | Sex-related differences in physiology, as well as in pharmacokinetics and pharmacodynamics of anesthetic drugs may influence the anesthesia plan, the management of pain, postoperative recovery, adverse effects (such a post-operative vomiting which is more common in women compared to men), patient satisfaction, and outcomes. However, further research on this topic is needed in how to optimize peri-operative care for female patients. All physicians should be aware of those differences, in peri-operative care, recovery and clinical care. |
| 4 | Urology and women | Women may experience urological health issues that are unique to their anatomy. Over the course of a lifetime, the body can change due to childbirth, menopause or surgeries in ways that could lead to conditions including pelvic floor weakness, overactive bladder, or inflammation of the bladder wall (interstitial cystitis). 35% of adult women struggle with urinary incontinence versus less than 7% of men, and women have a greater than 50% lifetime risk of urinary tract infections. Although bladder and kidney cancer are less common in women compared to men, these diagnosis should not be missed on the occasion that this is a more typical male diagnosis. Since urological issues are common in women and can be due to anatomical specific urology, it is important for all physicians to talk about these issues with your patients, mostly since they can be relatively easy to treat when caught early. |

| **Statement** | **References** |
| --- | --- |
| 1 | **Sleep Insomnia**  American Academy of Sleep Medicine and Sleep Research Society. (2015). [Recommended Amount of Sleep for a Healthy Adult: A Joint Consensus Statement of the American Academy of Sleep Medicine and Sleep Research Society](http://www.aasmnet.org/Resources/pdf/Adultsleepdurationconsensus.pdf) (PDF, 244 KB). *Journal of Clinical Sleep Medicine;* 11(6): 591–592.  Winkelman, J.W. (2015). [Insomnia Disorder](http://www.nejm.org/doi/full/10.1056/NEJMcp1412740) . *New England Journal of edicine;* 373: 1437-1444.  Mallampalli, M.P., Carter, C.L. (2014). [Exploring Sex and Gender Differences in Sleep Health: A Society for Women's Health Research Report](https://www.ncbi.nlm.nih.gov/pmc/articles/PMC4089020/). *Journal of Women's Health*; 23(7): 553-562. |
| 2 | **Auto-immune disease in women**  Invernizzi P, Pasini S, Selmi C, Gershwin ME, Podda M: Female predominance and X chromosome defects in autoimmune diseases. J Autoimmun. 2009, 33:12-16. 10.1016/j.jaut.2009.03.005  Angum F, Khan T, Kaler J, et al. (May 13, 2020) The Prevalence of Autoimmune Disorders in Women: A Narrative Review. Cureus 12(5): e8094. DOI 0.7759/cureus.8094  "Women at Risk" in Scientific American 325, 3, 40-45 (September 2021) doi:10.1038/scientificamerican0921-40 |
| 3 | **Peri-operative care in women**  Filipescu D, Ştefan M. Sex and gender differences in anesthesia: Relevant also for perioperative safety? Best Pract Res Clin Anaesthesiol. 2021 May;35(1):141-153. doi: 10.1016/j.bpa.2020.12.006. Epub 2020 Dec 8. PMID: 33742574.  Swisher J, Blitz J, Sweitzer B. Special Considerations Related to Race, Sex, Gender, and Socioeconomic Status in the Preoperative Evaluation: Part 2: Sex Considerations and Homeless Patients. Anesthesiol Clin. 2020 Jun;38(2):263-278. doi: 10.1016/j.anclin.2020.02.001. PMID: 32336383. |
|  | **Uroloy and women**  Institute of Medicine (IOM). (2011). Clinical preventive services for women: Closing the gaps. Washington, DC: The National Academies Press  Litwin M, Saigal C, Yano E, Avila C, Geschwind S, Hanley J, & Wang M (2005). Urologic Diseases In America Project: Analytical methods and principal findings. Journal of Urology, 173(3), 933–937.  Lucca I, Klatte T, Fajkovic H, de Martino M, Shariat SF. Gender differences in incidence and outcomes of urothelial and kidney cancer. Nat Rev Urol. 2015 Oct;12(10):585-92. doi: 10.1038/nrurol.2015.232. Erratum in: Nat Rev Urol. 2015 Dec;12(12):653. PMID: 26436686. |
